# Zn^2+^ Intoxication of Mycobacterium marinum during Dictyostelium discoideum Infection Is Counteracted by Induction of the Pathogen Zn^2+^ Exporter CtpC

**DOI:** 10.1128/mBio.01313-20

**Published:** 2021-02-02

**Authors:** Nabil Hanna, Hendrik Koliwer-Brandl, Louise H. Lefrançois, Vera Kalinina, Elena Cardenal-Muñoz, Joddy Appiah, Florence Leuba, Aurélie Gueho, Hubert Hilbi, Thierry Soldati, Caroline Barisch

**Affiliations:** a Department of Biochemistry, Faculty of Science, University of Geneva, Geneva, Switzerland; b Institute of Medical Microbiology, University of Zurich, Zurich, Switzerland; Institut Pasteur

**Keywords:** *Dictyostelium discoideum*, *Mycobacterium marinum*, infection, zinc poisoning, zinc transporters, ZnTs, CtpC

## Abstract

Macrophages use diverse strategies to restrict intracellular pathogens, including either depriving the bacteria of (micro)nutrients such as transition metals or intoxicating them via metal accumulation. Little is known about the chemical warfare between Mycobacterium marinum, a close relative of Mycobacterium tuberculosis (Mtb), and its hosts. We use the professional phagocyte Dictyostelium discoideum to investigate the role of Zn^2+^ during M. marinum infection. We show that M. marinum senses toxic levels of Zn^2+^ and responds by upregulating one of its isoforms of the Zn^2+^ efflux transporter CtpC. Deletion of *ctpC* (MMAR_1271) leads to growth inhibition in broth supplemented with Zn^2+^ as well as reduced intracellular growth. Both phenotypes were fully rescued by constitutive ectopic expression of the Mtb CtpC orthologue demonstrating that MMAR_1271 is the functional CtpC Zn^2+^ efflux transporter in M. marinum. Infection leads to the accumulation of Zn^2+^ inside the *Mycobacterium*-containing vacuole (MCV), achieved by the induction and recruitment of the D. discoideum Zn^2+^ efflux pumps ZntA and ZntB. In cells lacking ZntA, there is further attenuation of M. marinum growth, presumably due to a compensatory efflux of Zn^2+^ into the MCV, carried out by ZntB, the main Zn^2+^ transporter in endosomes and phagosomes. Counterintuitively, bacterial growth is also impaired in *zntB* KO cells, in which MCVs appear to accumulate less Zn^2+^ than in wild-type cells, suggesting restriction by other Zn^2+^-mediated mechanisms. Absence of CtpC further epistatically attenuates the intracellular proliferation of M. marinum in *zntA* and *zntB* KO cells, confirming that mycobacteria face noxious levels of Zn^2+^.

## Abstract (precis)

Microelements are essential for the function of the innate immune system. A deficiency in zinc or copper results in an increased susceptibility to bacterial infections.

## INTRODUCTION

Mycobacterium marinum is a pathogen that causes a tuberculosis-like infection in fish and amphibians, in which it generates granulomas very similar to those caused in humans by its close relative Mycobacterium tuberculosis (Mtb). M. marinum also opportunistically infects humans, but these infections are limited to the skin and extremities due to their lower temperature (1). At the level of the host macrophage, the course of infection by both M. marinum and Mtb is strikingly similar, making M. marinum an experimentally versatile model to study pathogenic mechanisms of tuberculosis (2). Apart from fish and mammalian phagocytes, another model host for M. marinum is the soil amoeba Dictyostelium discoideum (3), the endocytic and cell-autonomous defense pathways of which are well conserved with humans (4).

Upon phagocytic uptake, M. marinum resides inside a phagosome that is actively modified by the bacterium to rapidly divert from the canonical maturation pathway, to become a replicative niche, the so-called *Mycobacterium*-containing vacuole (MCV). Like Mtb, M. marinum was thought to be a vacuolar pathogen. However, accumulating evidence indicates that both bacteria also reside and proliferate in the cytosol, where access to nutrients is not limited but bacteria are exposed to cytosolic defenses (3, 5, 6). M. marinum starts damaging the MCV in the first hours of infection, before full rupture of the compartment releases the bacteria into the host cytosol at around 24 to 36 h. As also shown for Mtb, perforation of the MCV and escape to the cytosol are achieved, among other means, by secretion of the membrane-damaging peptide ESAT-6 through the mycobacterial ESX-1 type VII secretion system (T7SS) (3, 7–13).

Infected cells use a vast repertoire of strategies to fight intracellular mycobacteria. For instance, M. marinum infection has been shown to induce inducible nitric oxide synthase (iNOS) gene expression in fish macrophages (14), and neutrophils kill these bacilli through reactive oxygen species (ROS)-dependent mechanisms (15). In addition, M. marinum is targeted for digestion by the autophagy machinery of murine macrophages (16) and D. discoideum (3, 8). However, these mycobacteria have also evolved mechanisms of counterdefense against their hosts. They downregulate iNOS levels (17) and suppress the production of reactive nitrogen intermediates, decrease the expression of NADPH oxidase components, and reduce the production of ROS (14). Moreover, M. marinum avoids phagosome maturation by (i) modulating the composition of its cell wall (18) or the MCV content in phosphatidylinositol-3-phosphate (PtdIns3*P* [19]), (ii) impairing the recruitment of the endosomal sorting complex required for transport (ESCRT)-0 component Hrs (20), and (iii) avoiding accumulation of lysosomal enzymes (3, 16, 19, 21, 22). It also blocks the autophagic flux, which would eventually deliver the bacteria into autolysosomes for killing (3, 16).

Apart from the defense mechanisms mentioned above, little is known about the chemical warfare between M. marinum and its hosts, and especially about the manipulation of transition metals. In the case of Mtb, immune cells deprive the bacteria of essential nutrients such as iron and manganese, while they intoxicate the mycobacteria by accumulating copper and zinc inside the MCV (23–26). However, Mtb resists this metal-centered fight with an arsenal of metal-binding proteins, oxidases, and efflux transporters. For instance, Mtb captures Fe^3+^ from macrophages through its siderophore mycobactin (27), but it keeps low intracellular Cu^2+^ levels with its CtpV and MctB transporters and the Cu^2+^-binding metallothionein MymT (24, 28, 29). Mtb also resists the toxic free Zn^2+^ burst induced in human macrophages by exporting Zn^2+^ through the P-type ATPase CtpC (30), an efflux pump also present in two isoforms in M. marinum (31). On the host side, the mRNA levels of various Zn^2+^ transporters (i.e., ZIP8, ZnT1, ZIP1, and ZIP10) are altered in fish granulomas compared to resting macrophages (32). However, little is known about the conservation of these host-versus-pathogen strategies, and especially how the two mycobacterial CtpC and host Zn^2+^ transporters impact M. marinum infection in D. discoideum.

D. discoideum possesses four Zn^2+^ efflux transporters of the ZnT family (Znts) and 11 Zn^2+^ influx transporters of the ZIP family (Zpls). ZntA locates to the contractile vacuole (CV), an organelle that regulates the osmotic and metal balances in the cell, and ZntB to organelles of the endosomal pathway (33, 34). While ZntA does not have any close homologue, ZntB shares homology with human ZNT1 and ZNT10, the latter being also present at early endosomes (4). Similarly to their human homologs ZNT6 and ZNT7, located in the early secretory pathway (4), D. discoideum ZntC and ZntD localize to the Golgi complex or recycling endosomes (33). We wondered whether these ZnTs were part of the host defense mechanisms against M. marinum infection. We demonstrate here that D. discoideum intoxicates M. marinum by inducing the expression and recruitment of ZntA and ZntB, but not ZntC nor ZntD, to the MCV. However, M. marinum resists the D. discoideum Zn^2+^ immune response by specifically expressing its Zn^2+^ exporter CtpC.

## RESULTS

### Free Zn^2+^ accumulates within intact M. marinum-containing vacuoles.

**FIG 1:**
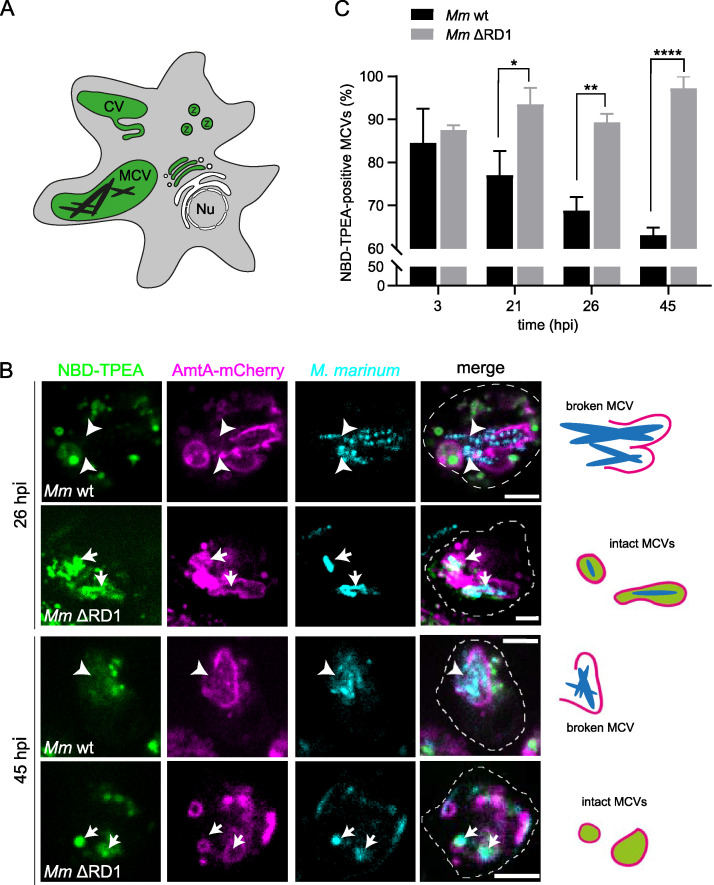
Free Zn^2+^ accumulates inside the MCV. (A) Scheme depicting the different localizations of free Zn^2+^ in D. discoideum: zincosomes (Z), contractile vacuole (CV), and phagosomes (e.g., MCVs [33]). Nu, nucleus. (B) Live cell imaging and illustrations of NBD-TPEA-treated cells expressing AmtA-mCherry (magenta). M. marinum was stained with Vybrant DyeCycle Ruby (cyan) before imaging. Arrows point at Zn^2+^-positive MCVs; arrowheads label bacteria exposed to the D. discoideum cytosol. Scale bars, 5 μm. One example is shown for wt and ΔRD1 M. marinum at 26 and 45 hpi, respectively. The cartoons illustrate the heterogeneity of Zn^2+^ labeling in intact and broken MCVs. (C) Percentage of NBD-TPEA-positive MCVs at different times post-infection with M. marinum wt and ΔRD1. Bars represent the mean and SEM from three independent experiments. About 300 wt and 200 ΔRD1 MCVs were counted in total. Statistical differences were calculated with a Fisher least significant difference (LSD) *post hoc* test after two-way ANOVA (*, *P* < 0.05; **, *P* < 0.01; ****, *P* < 0.0001).

In D. discoideum, free Zn^2+^ accumulates inside (i) the CV network, (ii) zincosomes (endosomal compartments with lysosomal and postlysosomal characteristics), and (iii) phagosomes containing beads and nonpathogenic bacteria such as Escherichia coli or Mycobacterium smegmatis (33, 35) (summarizing scheme is in Fig. 1A). We wondered whether vacuoles containing the pathogenic bacterium M. marinum or M. marinum ΔRD1, a mutant lacking a functional ESX-1 secretion system and thus strongly attenuated in inducing membrane damage and escaping to the cytosol (8, 9), also accumulate Zn^2+^. We monitored infected cells expressing the endosomal and MCV marker AmtA-mCherry (36) and labeled with the pH-independent Zn^2+^ probe NBD-TPEA (33) (Fig. 1B). Intact MCVs of the ΔRD1 mutant accumulated Zn^2+^ at all the stages of infection (Fig. 1B and C). In contrast, numerous compartments of M. marinum wild type (wt) appeared devoid of Zn^2+^, likely because it leaked out of either subtly or visibly broken MCVs (Fig. 1B). Quantification of NBD-TPEA-labeled MCVs confirmed that, because damage increases as infection progresses, fewer and fewer compartments containing wt bacteria were positive for Zn^2+^ (Fig. 1C).

### M. marinum senses and reacts to toxic levels of Zn^2+^ by inducing its CtpC Zn^2+^ efflux pump.

**FIG 2:**
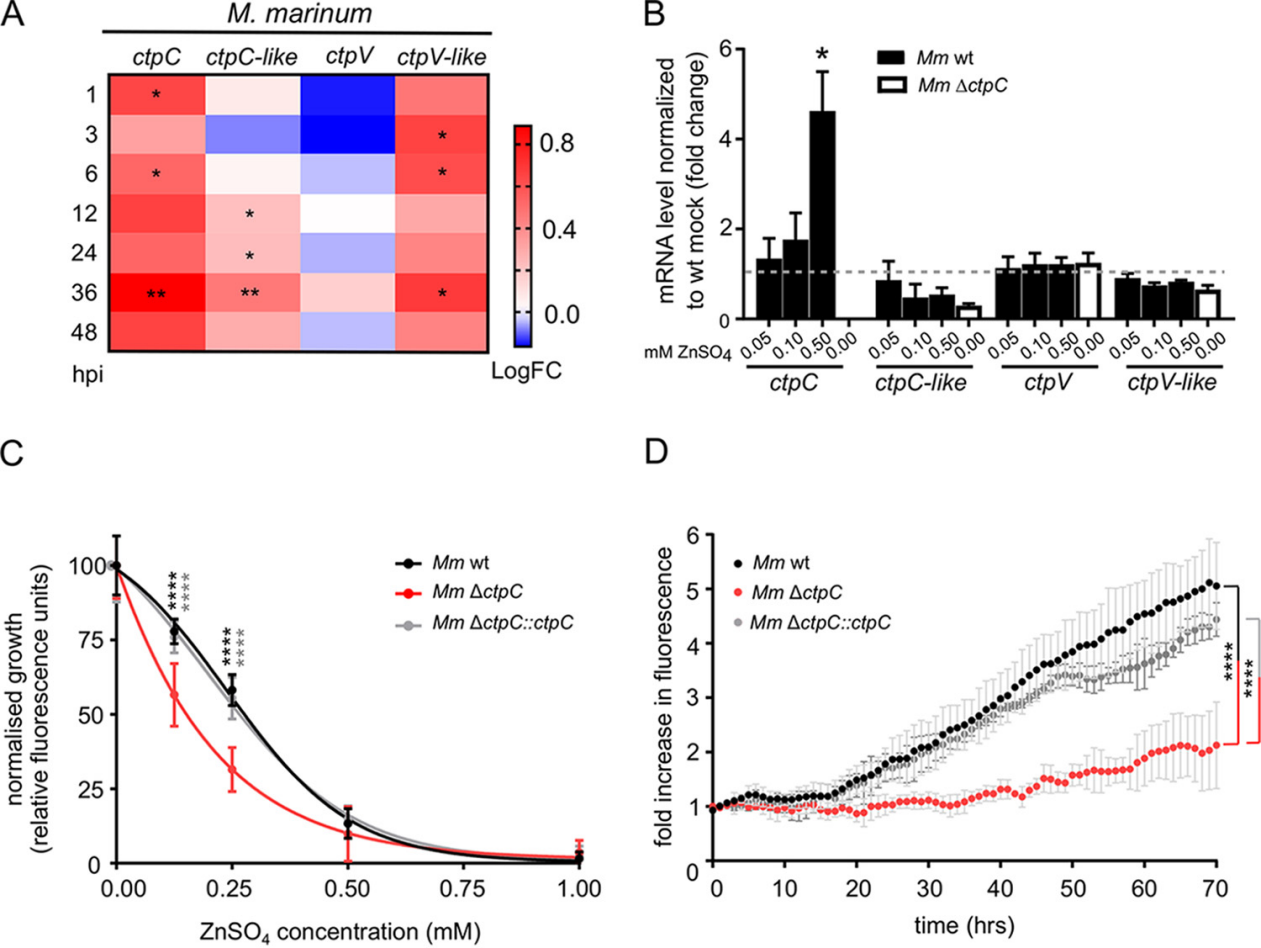
M. marinum senses and reacts to toxic levels of Zn^2+^ during infection and *in vitro*. (A) Heat map representing the transcriptional data shown in Table S1A. Cells were infected with GFP-expressing M. marinum wt, and samples were collected at different hpi. The time points with statistically significant differential expression are marked with asterisks (*, *P* < 0.05; **, *P* < 0.01). Colors indicate the amplitude of expression of M. marinum *ctpC*, *ctpC*-like, *ctpV*, and *ctpV*-like (in logarithmic fold change [logFC]) in infected cells compared to M. marinum grown in broth: from dark red (highest expression) to dark blue (lowest expression). (B) Normalized mRNA levels of *Mm*_*ctpC*, *Mm_ctpC*-like, *Mm*_*ctpV*, and *Mm_CtpV*-like in GFP-expressing M. marinum grown in 7H9 with increasing concentrations of ZnSO_4_ compared to bacteria grown in 7H9 without extra ZnSO_4_ (depicted as dashed gray line). To confirm the successful deletion of *ctpC*, the mRNA levels in M. marinum Δ*ctpC* were tested as well. Shown are mean and standard deviations from two independent experiments. Statistical differences were calculated with an unpaired *t* test (*, *P* < 0.05). (C) Dose-response curves of M. marinum wt, Δ*ctpC*, and Δ*ctpC*::*ctpC* grown in broth supplemented with increasing concentrations of ZnSO_4_ (0, 0.125, 0.25, 0.5, and 1 mM). The fluorescence intensities of GFP and E2-Crimson were used as a proxy for bacterial growth. The areas under the curve were calculated for the three strains, and the values were plotted as a function of ZnSO_4_ concentrations. Error bars indicate the SD from eight technical replicates from two independent biological replicates. Statistical differences were calculated with a Fisher LSD test after two-way ANOVA (****, *P* < 0.0001). (D) D. discoideum was infected with GFP- or E2-Crimson-expressing M. marinum wt, Δ*ctpC* (GFP expressing), or Δ*ctpC*::*ctpC* (E2-Crimson expressing). The intracellular bacterial growth (in relative fluorescence units [RFUs]) was monitored every hour. Shown is the fold increase in bacterial fluorescence over time of two independent biological replicates. Error bars indicate the SD from eight technical replicates. Statistical differences were calculated with Dunnett’s multiple-comparison test after two-way ANOVA (****, *P* < 0.0001).

During infection of macrophages, Mtb is exposed to a burst of transition metals that induces the expression of efflux P-type ATPases such as CtpC, CtpG, and CtpV (30, 37). In particular, CtpC expression helps Mtb detoxify excess Zn^2+^, which the host transports inside the MCV (30). The expression of Mtb *ctpV* is also induced during macrophage infection (28), which is essential for virulence and survival in response to elevated copper levels in the MCV (28). The genome of M. marinum encodes 20 P-type ATPases that are phylogenetically close to their Mtb homologues (see Fig. S1 in the supplemental material). Interestingly, M. marinum has two homologues of CtpC, CtpC (MMAR_1271) and CtpC-like (MMAR_2147). Inspection of our parallel RNA-sequencing (RNA-Seq) results of M. marinum and D. discoideum during infection (38) revealed that, as shown for Mtb, expression of the *ctpC* gene increased at all time points and reached its maximum 36 h postinfection (hpi) compared to M. marinum grown in broth (Fig. 2A, Fig. S2, and Table S1A). Expression of the *ctpc*-like gene was increased only later during infection and also peaked at 36 hpi (Fig. 2A, Fig. S2, and Table S1A). These results suggest a possible specific role of the two CtpC P-type ATPases in the detoxification of Zn^2+^ during infection. It is noteworthy that other P-type ATPases were differentially expressed during infection, including the three M. marinum CtpV homologues, suggesting a complex scenario in which M. marinum faces elevated levels of other ions (Fig. S2 and Table S1A).

10.1128/mBio.01313-20.1 FIG S1 Phylogenetic tree of P-type ATPases of Mtb and M. marinum. Putative P-type ATPases of M. marinum were identified on Mycobrowser (66) and aligned with MAFFT to generate a phylogenetic tree. Download FIG S1, PDF file, 0.03 MB. Copyright © 2021 Hanna et al. 2021 Hanna et al. This content is distributed under the terms of the Creative Commons Attribution 4.0 International license.

10.1128/mBio.01313-20.2 FIG S2 Differential expression of M. marinum P-type ATPases during infection of *Dictyostelium*. Heat map representing the transcriptional data shown in Table S1A. Cells were infected with GFP-expressing M. marinum wt, and samples were collected at different hpi. The time points with statistically significant differential expression are marked with asterisks (*, *P* < 0.05; **, *P* < 0.01). Colors indicate the differential expression of P-type ATPases (in logFC) in infected cells compared to bacteria that were grown in 7H9 medium: from dark red (highest expression) to dark blue (lowest expression). Download FIG S2, PDF file, 0.2 MB. Copyright © 2021 Hanna et al. 2021 Hanna et al. This content is distributed under the terms of the Creative Commons Attribution 4.0 International license.

10.1128/mBio.01313-20.8 TABLE S1 Differential expression of M. marinum P-type ATPases (A), D. discoideum CV genes (B), D. discoideum *zntA* to *zntD* transporters (C), and D. discoideum *zplA* to *zplG* transporters (D) during infection. D. discoideum wt was either infected with GFP-expressing M. marinum wt or mock infected. Samples were collected at different times postinfection (1, 3, 6, 12, 24, 36, and 48 hpi) and sorted for enrichment in infected cells (see Materials and Methods). Shown are the logarithmic fold changes (LogFC) in expression between infected and mock-infected samples (for D. discoideum genes) or between intracellular M. marinum and bacteria grown in broth (for M. marinum genes). Data extracted from reference 38. Download Table S1, DOCX file, 0.03 MB. Copyright © 2021 Hanna et al. 2021 Hanna et al. This content is distributed under the terms of the Creative Commons Attribution 4.0 International license.

We next attempted to evaluate whether the expression of *ctpC* is influenced by Zn^2+^. To this end, the expression levels of selected P-type ATPases were determined by qRT-PCR analysis. Supplementing the growth medium with increasing concentration of ZnSO_4_ strongly enhanced the transcription of *ctpC* but not *ctpC*-like, *ctpV*, or *ctpV*-like (Fig. 2B). These results suggest that CtpC is exclusively involved in detoxification of Zn^2+^ in M. marinum and that CtpC-like might function under different circumstances than those tested.

To investigate the physiological role of CtpC, we constructed a deletion mutant of *ctpC* (MMAR_1271; Fig. 2B). M. marinum wt and its isogenic mutant Δ*ctpC* grew similarly in broth and on agar, indicating no constitutive growth defect (Fig. S3A and E to G). However, the growth of M. marinum Δ*ctpC* in broth was impaired in the presence of Zn^2+^ in a dose-dependent manner (Fig. 2C and Fig. S3B to E). In addition, its growth was impaired on solid medium containing elevated levels of Zn^2+^ but not of Mn^2+^ or Cu^2+^ (Fig. S3E to G), demonstrating that the absence of *ctpC* renders M. marinum specifically susceptible to Zn^2+^. Strikingly, the intracellular growth of M. marinum Δ*ctpC* was notably impaired (Fig. 2D). Most importantly, both the growth defect of Δ*ctpC* in broth supplemented with Zn^2+^ (Fig. 2C) and the intracellular growth (Fig. 2D) were fully rescued by constitutive expression of the Mtb CtpC orthologue (Rv3270). Together, these results demonstrate that the susceptibility of M. marinum Δ*ctpC* to Zn^2+^ is solely due to the lack of CtpC and that MMAR_1271 is the functional orthologue of Mtb CtpC. We conclude that, during infection of D. discoideum, M. marinum is exposed to toxic concentrations of Zn^2+^ and that the bacteria counteract this intoxication by specifically inducing the Zn^2+^ efflux pump CtpC.

10.1128/mBio.01313-20.3 FIG S3 High concentrations of Zn^2+^ inhibit M. marinum Δ*ctpC* growth in broth and on agar whereas Mn^2+^ and Cu^2+^ have no differential effect on growth. (A to D) Growth of GFP- or E2-Crimson-expressing M. marinum wt, Δ*ctpC* (GFP expressing), and Δ*ctpC*::*ctpC* (E2-Crimson expressing) was assessed in broth by measuring the increase of fluorescence. Shown is the fold increase in bacterial fluorescence over time for two independent biological replicates. Error bars indicate the SD from eight technical replicates. Statistical differences of pairwise comparisons were calculated with a Fisher LSD *post hoc* test after two-way ANOVA (***, *P* < 0.001; ****, *P* < 0.0001). (E to G) GFP-expressing M. marinum wt and Δ*ctpC* were plated on 7H11 or 7H11 supplemented with increasing concentrations of ZnSO_4_ (E), MnCl_2_ (F), or CuSO_4_ (G) (0, 0.125, 0.25, 0.5, 1, and 2.5 mM) as described in Materials and Methods. Shown are the bacterial colonies obtained in one representative of three independent biological replicates, after 7 days of incubation at 32°C. Download FIG S3, PDF file, 0.9 MB. Copyright © 2021 Hanna et al. 2021 Hanna et al. This content is distributed under the terms of the Creative Commons Attribution 4.0 International license.

### The D. discoideum Zn^2+^ transporters ZntA and ZntB localize to the M. marinum MCV.

**FIG 3:**
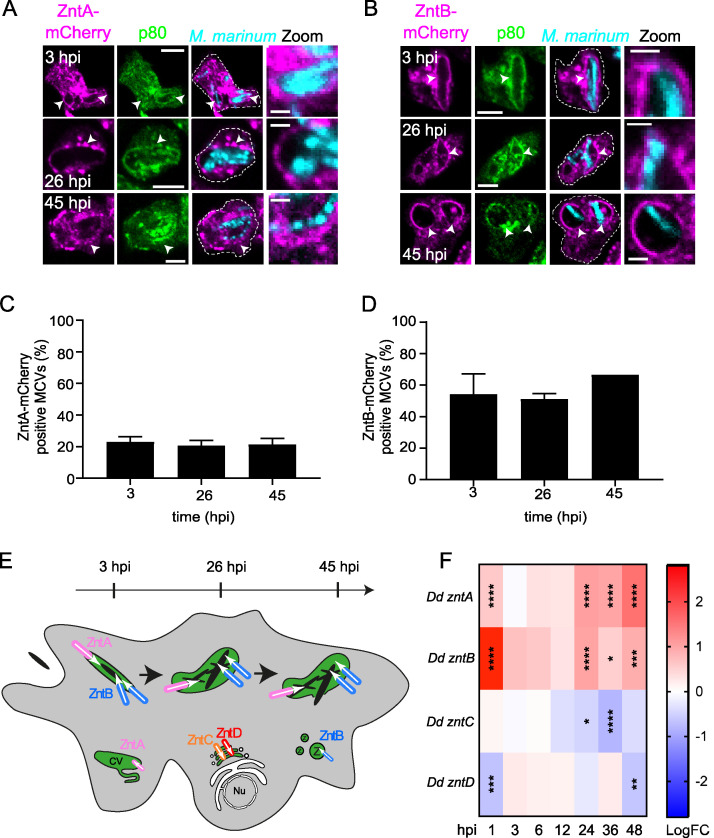
ZntA and ZntB transporters are recruited to the MCV and induced upon infection. (A and B) Immunofluorescence staining at different times postinfection of ZntA- or ZntB-mCherry-expressing cells (shown in magenta) infected with GFP-expressing M. marinum (shown in cyan). MCVs were visualized by staining for the endocytic marker p80 (21) (shown in green). Arrowheads mark Znt-positive MCVs. Scale bars, 5 μm (zoom, 2 μm). (C and D) Quantification of panels A and B, respectively. Two independent experiments were performed. 305 and 246 MCVs were analyzed manually for the presence of ZntA and ZntB at MCVs, respectively. (E) Scheme depicting the localization of the four D. discoideum Znts during infection with M. marinum. CV, contractile vacuole; Nu, nucleus. (F) Heat map representing the transcriptional data shown in Table S1C. Cells were either infected with GFP-expressing M. marinum wt or mock infected, and samples were collected at different hpi. The time points with statistically significant differential expression are marked with asterisks (*, *P* < 0.05; **, *P* < 0.01; ***, *P* < 0.001; ****, *P* < 0.0001). Colors indicate the strength of expression of *zntA* to *zntD* (in logFC) in infected cells compared to mock infected: from dark red (highest expression) to dark blue (lowest expression).

We wondered which of the four D. discoideum Zn^2+^ efflux transporters ZntA, ZntB, ZntC, and ZntD (33) localized to the MCV and led to Zn^2+^ accumulation. To ensure optimal preservation of membranes and organelles including the MCV and CV for immunofluorescence, we performed fixation/permeabilization in ultracold methanol (39, 40). Only ZntA and ZntB (Fig. 3A and B), but neither ZntC nor ZntD (Fig. S4A and B), localized to the MCV. While 20% of the MCVs were positive for ZntA (Fig. 3C), ZntB was present at around 50 to 60% of the MCVs throughout the infection cycle (Fig. 3D). Because ZntA is localized exclusively at the CV membrane in noninfected cells (33), we confirmed its localization at the MCV by live imaging. Strikingly, besides ZntA (Fig. S4C) other CV resident proteins such as Rab11a and Rab11c (41) were present at the MCV (Fig. S4D and E), emphasizing a possible cross talk between the two organelles. CV proteins were also found to be enriched in the MCV proteome (Fig. S4F and Table S2) (42). In addition, differential expression of genes encoding many CV proteins (38) suggests a role for this organelle during infection (Fig. S4F and Table S1B) (38).

10.1128/mBio.01313-20.4 FIG S4 ZntC and ZntD transporters do not localize at the MCV. (A and B) Immunofluorescence staining at different times postinfection of ZntC- or ZntD-mCherry-expressing cells (shown in magenta) infected with GFP-expressing M. marinum (shown in cyan). MCVs were visualized by staining for p80 (green). Arrows point to ZntC/D-negative MCVs. Scale bars, 5 μm. (C to E) Live imaging of ZntA-mCherry-, Rab11a-RFP-, and Rab11c-RFP-expressing cells (shown in magenta) infected with GFP-expressing M. marinum (shown in cyan). Images were taken at the indicated time points. Two examples are for shown for ZntA-, Rab11a-, or Rab11c-positive MCVs at 26 or 21 hpi, respectively. Arrowheads point to MCVs decorated with ZntA-mCherry, Rab11a-RFP, or Rab11c-RFP. Scale bars, 5 μm. (F) Enrichment of D. discoideum CV proteins at the MCV and their differential expression during M. marinum infection. Table showing the presence of selected CV proteins in the MCV proteome. Data were retrieved from Table S1 of the work of Gueho et al. (42). Information of the MCV enrichment of all 37 CV proteins (GO-term: CV) is presented also in Table S2. Heat map representing the transcriptional data shown in Table S1B. Cells were either infected with GFP-expressing M. marinum wt or mock infected, and samples were collected at different hpi. The time points with statistically significant differential expression are marked with asterisks (*, *P* < 0.05; **, *P* < 0.01; ***, *P* < 0.001; ****, *P* < 0.0001). Colors indicate the strength of expression (in logFC) in infected cells compared to mock infected: from dark red (highest expression) to dark blue (lowest expression). Download FIG S4, PDF file, 0.7 MB. Copyright © 2021 Hanna et al. 2021 Hanna et al. This content is distributed under the terms of the Creative Commons Attribution 4.0 International license.

10.1128/mBio.01313-20.9 TABLE S2 Enrichment of CV proteins at the MCV. Analysis of recent proteomic data of early M. marinum MCVs (Table S1 of reference 42) reveals a possible cross-talk between the CV and MCV. Thirty-seven proteins annotated on dictyBase with the GO term “Contractile Vacuole” were analyzed for their presence in the MCV proteome. For more information, please see the work of Guého et al. (42). Download Table S2, DOCX file, 0.01 MB. Copyright © 2021 Hanna et al. 2021 Hanna et al. This content is distributed under the terms of the Creative Commons Attribution 4.0 International license.

10.1128/mBio.01313-20.10 TABLE S3 D. discoideum and M. marinum material used for this study. (A) D. discoideum material used for this study. (B) M. marinum material used for this study. Download Table S3, DOCX file, 0.03 MB. Copyright © 2021 Hanna et al. 2021 Hanna et al. This content is distributed under the terms of the Creative Commons Attribution 4.0 International license.

In summary, the data suggest that ZntB is the main MCV Zn^2+^ transporter but that ZntA might also contribute to the import of Zn^2+^ into the MCV (summarizing scheme is in Fig. 3E).

### The expression of *Dictyostelium* ZnTs is altered during infection with M. marinum.

M. marinum infection in zebrafish modulates the expression of various Zn^2+^ transporters in granulomas (32). Further inspection of our dual RNA-Seq results of D. discoideum during infection with M. marinum (38) showed that the expression of *zntA* and *zntB* was upregulated at very early, mid-, and late stages of infection, with the highest levels found for *zntB* at 1 hpi (Fig. 3F and Table S1C) (38). In contrast, the expression of *zntC* and *zntD* was not or only weakly affected. We conclude that expression of ZntA and ZntB increases during M. marinum infection and also that their localization at the MCV contributes to the accumulation of Zn^2+^ inside the compartment. On the other hand, ZntC and ZntD, which are absent from the MCV, either are not relevant or might only play an indirect role by contributing to an accumulation of Zn^2+^ in other organelles that are linked to the MCV by membrane transport.

### Knockout of ZntA and ZntB impact on the concentration of Zn^2+^ inside the MCV.

**FIG 4:**
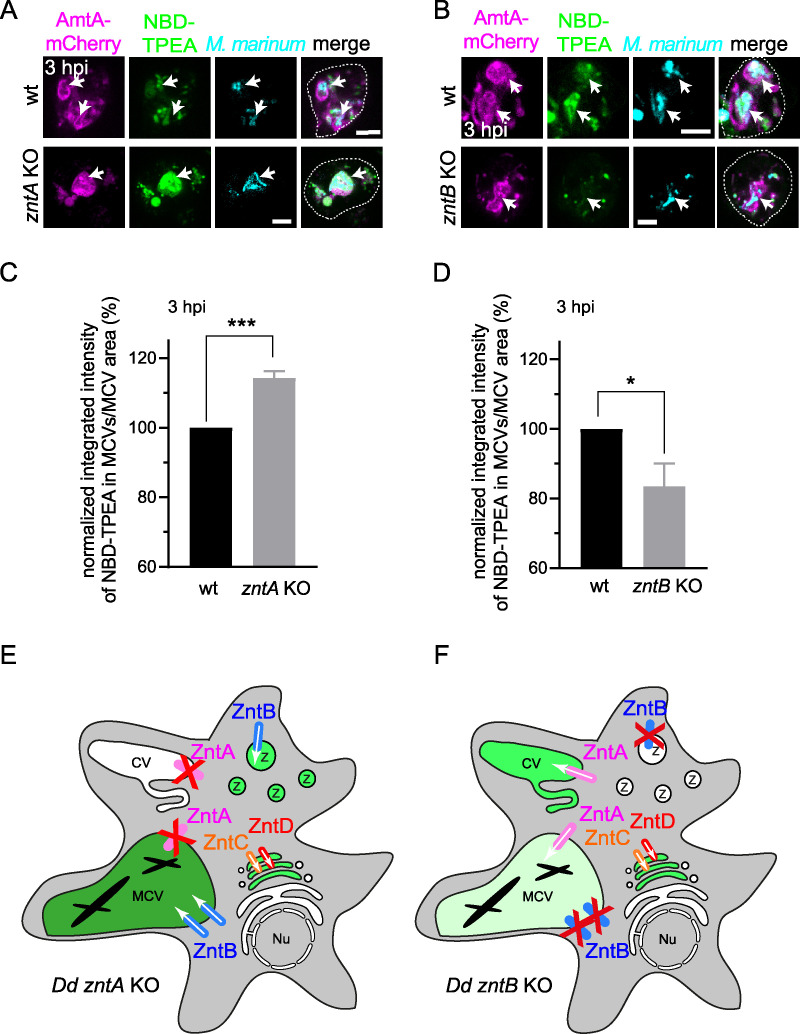
Zn^2+^ accumulates in MCVs of cells lacking ZntA but decreases in *zntB* KO amoebae. (A and B) Live imaging of NBD-TPEA-treated wt and *zntA* KO cells (A) or *zntB* KO cells (B) expressing AmtA-mCherry (magenta). M. marinum was stained with Vybrant DyeCycle Ruby (cyan) before imaging at 3 hpi (B) or 46 hpi (A). Arrows label MCVs. Scale bars, 5 μm. (C and D) Quantification of the normalized integrated intensity of NBD-TPEA inside MCVs per MCV area at 3 hpi in Ax2(Ka) wt and *zntA* KO cells (C) or Ax4 wt and *zntB* KO cells (D) infected with mCherry-expressing M. marinum. Images at 3 hpi were taken automatically using an ImageXpress spinning disc confocal microscope (Molecular Devices). To quantify NBD-TPEA inside MCVs, a MetaXpress (Molecular Devices) pipeline that detects NBD-TPEA inside MCVs was set up (see Materials and Methods). Four independent experiments were performed. A total of 7,353, 8,559, 5,713, and 4,679 MCVs were analyzed in infected Ax2(Ka) wt, Ax2(Ka) *zntA* KO, Ax4 wt, and Ax4 *zntB* KO cells, respectively. Statistical differences were assessed with an unpaired *t* test (*, *P* < 0.05; ***, *P* < 0.001). (E and F) Summarizing schemes illustrating the mislocalization of Zn^2+^ in infected *zntA* (E) or *zntB* (F) KO cells. While depletion of ZntA leads to an accumulation of Zn^2+^ into zincosomes and the MCV, loss of ZntB leads to a reduction of Zn^2+^ inside MCVs. Z, zincosomes; CV, contractile vacuole; MCV, *Mycobacterium*-containing vacuole; Nu, nucleus.

We had previously shown that in the absence of ZntA, lumenal Zn^2+^ increases in acidic zincosomes and phagosomes, while in cells lacking ZntB, zincosomes contain low levels of Zn^2+^ (33). To determine the relevance of ZntA and ZntB in the restriction of M. marinum by Zn^2+^, we infected cells lacking one or the other transporter and monitored the levels of Zn^2+^ inside early, intact MCVs (Fig. 4A to D). Similarly to bead-containing phagosomes, MCVs accumulated more Zn^2+^ in *zntA* knockout (KO) cells while the levels of Zn^2+^ were lower in MCVs from *zntB* KO cells than from wt cells (Fig. 4A to D). This suggests that ZntB, the main lysosomal and postlysosomal Zn^2+^ transporter (33), might compensate for the absence of the main CV efflux detoxifier ZntA (33) by exporting Zn^2+^ into all endosomes and MCVs (summarizing schemes are in Fig. 4E and F).

### Zn^2+^ restricts M. marinum during infection.

Accumulation of higher Zn^2+^ levels in phagosomes of D. discoideum *zntA* KO cells results in a more efficient killing of the nonpathogenic bacterium E. coli (33). Plaque formation by D. discoideum on lawns of “food” bacteria spiked with pathogenic bacteria allows monitoring of either the virulence of the pathogen or the resistance/susceptibility of D. discoideum to these pathogens (43). Monitoring of plaque formation on lawns containing M. marinum confirmed that the *zntA* KO was less affected by the presence of M. marinum than wt D. discoideum (Fig. S5A and B). Interestingly, both D. discoideum wt and *zntA* KO grew better on lawns containing the attenuated M. marinum Δ*ctpC* mutant (Fig. S5A and B), confirming a Zn^2+^-dependent defense process. In contrast, D. discoideum *zntB* KO cells did not benefit from a growth advantage on M. marinum-containing lawns (Fig. S5C and D), corroborating that the uptake, killing, and digestion of food bacteria are unaffected in these cells, although they have lower lumenal Zn^2+^ concentrations. Importantly, plaque formation on a variety of nonpathogenic bacteria was comparable for *zntA* KO, *zntB* KO, and wt cells (Fig. S5E and F). We conclude that the resistance of D. discoideum is proportional to both the level of Zn^2+^ within the MCV (which is higher in the *zntA* KO) and the capacity of M. marinum to detoxify its own intracellular Zn^2+^ (33).

10.1128/mBio.01313-20.5 FIG S5 Growth of D. discoideum z*ntA* KO or *zntB* KO cells on M. marinum lawns. (A to D) Plaque formation by Ax2(Ka) wt and *zntA* KO (A) or Ax4 wt and *zntB* KO cells (C) on GFP-expressing M. marinum wt or Δ*ctpC*, as described in Materials and Methods. Shown is one representative experiment from three independent biological replicates. (B and D) Quantification of three independent experiments. Plaquing score was calculated as described in Materials and Methods. (E and F) Plaque formation by Ax2(Ka) wt and *zntA* KO (E) or Ax4 wt and *zntB* KO cells (F) on nonpathogenic bacteria, as described in Materials and Methods. Download FIG S5, PDF file, 0.2 MB. Copyright © 2021 Hanna et al. 2021 Hanna et al. This content is distributed under the terms of the Creative Commons Attribution 4.0 International license.

**FIG 5:**
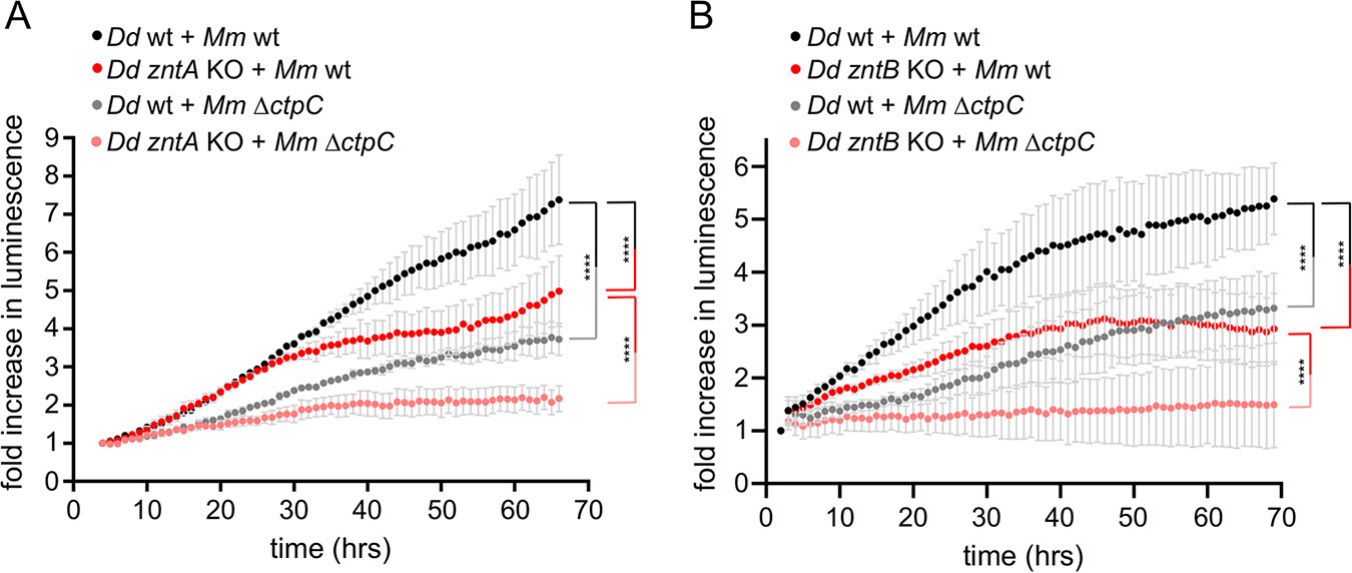
M. marinum intracellular growth is impaired in cells lacking ZntA or ZntB. Ax2(Ka) wt or *zntA* KO cells (A) and Ax4 wt or *zntB* KO cells (B) were infected with luciferase-expressing M. marinum wt or Δ*ctpC*, and the intracellular bacterial growth (in RLUs) was monitored every hour. Shown is the fold increase in bacterial luminescence over time. Error bars indicate the SEM from three independent experiments. Statistical differences of pairwise comparisons were calculated with a Fisher LSD *post hoc* test after two-way ANOVA (****, *P* < 0.0001).

Next, we monitored the impact of *znt* KOs on M. marinum intracellular growth. Strikingly, absence of ZntA led to a decrease in the intracellular load of M. marinum (Fig. 5A), suggesting that the bacteria encounter noxious levels of Zn^2+^ during infection (Fig. 4A and C). This was further corroborated by the fact that compared to M. marinum wt, the intracellular load of M. marinum Δ*ctpC*, which is more sensitive than wt to high Zn^2+-^levels (Fig. 2C), was decreased in *zntA* KO cells (Fig. 5A). This epistatic interaction between M. marinum Δ*ctpC* and D. discoideum *zntA* KO (i.e., the higher growth restriction of Δ*ctpC* in the *zntA* KO compared to wt host) is strong evidence for the role of Zn^2+^ during infection.

Intriguingly, although the concentration of Zn^2+^ inside the MCVs was lower in cells lacking ZntB (Fig. 4B and D), the intracellular bacterial load also decreased with time in these mutant cells (Fig. 5B), comparably to what happens inside *zntA* KO cells (Fig. 5A). But we also noticed that, while bacterial growth leveled off inside *zntA* KO cells only after 24 hpi (Fig. 5A), the growth of M. marinum in *zntB* KO cells was impaired from the beginning of the infection (Fig. 5B). One possible explanation is that phagosomal escape of M. marinum is altered in the *zntB* KO cells, exposing them either to a stronger restriction by cytosolic defenses in the case of an earlier escape (9, 44) or to a prolonged restriction by vacuolar Zn^2+^ is the case that MCV escape is inhibited.

To assess whether the MCV integrity is compromised in D. discoideum lacking *zntA* or *zntB*, we monitored selected readouts for MCV membrane damage and M. marinum cytosolic access (36, 44). In brief, membrane damage induced by M. marinum leads to bacterial and MCV ubiquitination and recruitment of the autophagy machinery (44). Consequently, the fraction of ubiquitin-positive mycobacteria is a proxy for damage. In addition, Plin, the D. discoideum homologue of mammalian perilipins, binds the hydrophobic mycobacterial cell wall when the mycobacteria are exposed to the cytosol (36). Interestingly, upon infection with M. marinum wt, the fractions of bacteria ubiquitinated or associated with Plin were comparable in all host cells (Fig. S6A to D), suggesting that membrane damage but also the detection of cytosolic bacteria is not affected in these mutants. In addition, no significant difference in ubiquitination was observed for the Δ*ctpC* M. marinum mutant, neither in wt nor in the *zntA* and *zntB* KO cells (Fig. S6A and B). However, a strong reduction of Plin-positive Δ*ctpC* bacteria was observed in *zntB* KO cells, suggesting that Δ*ctpC* might escape less efficiently to the cytosol in these cells (Fig. S6D). Nevertheless, the epistatic interaction observed between M. marinum Δ*ctpC* and D. discoideum *zntB* KO during the assessment of intracellular growth (Fig. 5B) suggests that Zn^2+^ plays a major role in the restriction of these bacteria also in the absence of D. discoideum ZntB.

10.1128/mBio.01313-20.6 FIG S6 Membrane damage and phagosomal escape of M. marinum wt and Δ*ctpC* in D. discoideum *zntA* and *zntB* KO cells. (A and B) Ax2(Ka) wt or *zntA* KO cells (A) and Ax4 wt or *zntB* KO cells (B) were infected with GFP-expressing M. marinum wt and Δ*ctpC*. The percentage of ubiquitinated bacteria was assessed by immunofluorescence using FK2 antibodies and manual counting at 26 hpi. A total of 106, 95, 95, and 68 bacteria were counted in Ax2(Ka) wt, Ax2(Ka) *zntA* KO, Ax4 wt, and *zntB* KO cells infected with M. marinum wt, respectively, and a total of 115, 91, 94, and 86 bacteria were counted in Ax2(Ka) wt, *zntA* KO, Ax4 wt, and *zntB* KO cells infected with M. marinum Δ*ctpC*, respectively. Unpaired *t* tests showed no statistical differences. (C and D) Ax2(Ka) wt or *zntA* KO cells (C) and Ax4 wt or *zntB* KO cells (D) expressing mCherry-Plin were infected with GFP-expressing M. marinum wt and Δ*ctpC*. The percentage of cells with intracellular bacteria colocalizing with Plin was assessed manually at 26 hpi. A total of 178, 136, 85, and 29 bacteria were counted in Ax2(Ka) wt, *zntA* KO, AX4 wt, and *zntB* KO cells infected with M. marinum wt, respectively, and a total of 96, 85, 65, and 19 bacteria were counted in Ax2(Ka) wt, *zntA* KO, Ax4 wt, and *zntB* KO cells infected with M. marinum Δ*ctpC*, respectively. Error bars indicate the SEM from four (C) or three (D) independent experiments. Statistical differences are indicated with an asterisk and were calculated with an unpaired *t* test (*, *P* < 0.05). Download FIG S6, PDF file, 0.06 MB. Copyright © 2021 Hanna et al. 2021 Hanna et al. This content is distributed under the terms of the Creative Commons Attribution 4.0 International license.

## DISCUSSION

Upon bacterial invasion, immune phagocytes manipulate their intracellular Zn^2+^ pools for nutritional immunity and/or metal intoxication purposes. Accordingly, infected neutrophils reduce Streptococcus pyogenes growth by releasing the Zn^2+^ scavenger calprotectin (45), while they enhance phagosomal Zn^2+^ levels for bacterial intoxication (46). Calprotectin also protects cells against other bacteria such as Helicobacter pylori (47), Borrelia burgdorferi (48), or Staphylococcus aureus (49). Using a different strategy, macrophages enhance bacterial clearance by delivering Zn^2+^ to E. coli*-*, Salmonella enterica serovar Typhimurium-, and Mtb-containing compartments (30, 50). However, pathogens have evolved mechanisms to counteract Zn^2+^-mediated defenses. For instance, *Salmonella* evades Zn^2+^-containing vesicles by means of its pathogenicity island 1 (SPI-1) (50), while Mtb exports Zn^2+^ through its P-type ATPase CtpC (30). In addition, upon calprotectin-mediated Zn^2+^ chelation, *Salmonella* induces the expression of its Zn^2+^ importer ZnuABC (51). In this study, we focused on the Zn^2+^-mediated battle between M. marinum and its experimental host D. discoideum.

D. discoideum is a model to study the role of transition metals in the fight against intracellular pathogens (35, 52–54). For example, while Fe^2+^ participates in the defense against Mycobacterium avium or Legionella pneumophila (35, 52–54), Cu^2+^ or Zn^2+^ does not affect the infection by *Legionella* (35). On the other hand, to access poorly available transition metals such as iron, M. marinum stimulates the synthesis and release of small chelating molecules known as siderophores (55).

In contrast to the 24 transporters encoded in humans, D. discoideum possesses only 11 Zn^2+^ transporters: seven ZIP-like proteins (ZplA to -G) and four ZnTs (ZntA to -D) (4). ZntA is the main transporter in the CV and a key regulator of Zn^2+^ homeostasis in D. discoideum. The transporter is not found in the endocytic and phagocytic pathways in noninfected cells. When ZntA is knocked out, cells compensate for its absence by increasing pumping of Zn^2+^ inside the compartments of the endocytic pathway through the other transporters ZntB to ZntD (33). ZntB is the main Zn^2+^ importer in lysosomes and recycling endosomes (33). Interestingly, throughout the intracellular infection cycle of M. marinum, both ZntA and ZntB were located at MCVs (Fig. 3A to E), although at different levels. While ZntA localized at detectable levels only to a minor fraction of MCVs (around 20% [Fig. 3C]), ZntB was present at the majority of them (50 to 60% [Fig. 3D]). This, together with the fact that the expression of ZntB notably increased during M. marinum infection (Fig. 3F) and that MCVs accumulate less Zn^2+^ in cells lacking ZntB (Fig. 4B, D, and F), led us to hypothesize that ZntB is the main Zn^2+^ efflux transporter during infection by M. marinum, directly pumping Zn^2+^ into the MCVs. Since accumulation of Zn^2+^ within MCVs was not completely abolished upon ZntB depletion, we propose that Zn^2+^ can also be transferred into MCVs by direct transport via ZntA, especially considering that the levels of the *zntA* mRNA increase at all stages of infection (Fig. 2A). In addition, fusion of MCVs with zincosomes, as shown to occur to the M. smegmatis-containing compartment (33), might also contribute to elevated lumenal Zn^2+^ levels. As ZntC and ZntD are localized to other compartments of the endocytic and secretory pathways, they could indirectly contribute to lumenal Zn^2+^ levels (33). But a direct role of these transporters can likely be disregarded, because ZntC and ZntD were not detected at the MCV at any time postinfection (Fig. 3E and see also Fig. S4A and B in the supplemental material), and the levels of their mRNA were not altered, or only slightly reduced, during infection with M. marinum (Fig. 3F and Table S1C). Previous studies have shown that the expression of ZNT1, the homologue of ZntB, increases in human macrophages after 18 and 72 h of infection with Mtb (26, 30, 32). This suggests that D. discoideum reacts to the invasion by pathogenic mycobacteria in a manner similar to mammalian macrophages.

The complex Zn^2+^ homeostasis is regulated by the balance of ZnT efflux transporters and ZIP influx transporters. In other words, higher ZIP-mediated pumping of Zn^2+^ to the cytosol is compensated for by detoxification, via ZnT-mediated import of Zn^2+^ inside various compartments, including the MCV. On the other hand, decreased ZIP-mediated transport from endosomes to the cytosol might also increase lumenal Zn^2+^. In keeping with these scenarios, the expression of many of the D. discoideum Zpl influx pumps is affected in complex manners during infection with M. marinum (Fig. S7 and Table S1D).

10.1128/mBio.01313-20.7 FIG S7 Differential expression of the D. discoideum ZIP transporters during M. marinum infection. Heat map representing the transcriptional data shown in Table S1D. Cells were either infected with GFP-expressing M. marinum wt or mock infected, and samples were collected at different hpi. The time points with statistically significant differential expression are marked with asterisks (*, *P* < 0.05; **, *P* < 0.01; ***, *P* < 0.001; ****, *P* < 0.0001). Colors indicate the strength of expression of *zplA* to -*G* (in logFC) in infected cells compared to mock infected: from dark red (highest expression) to dark blue (lowest expression). Download FIG S7, PDF file, 0.04 MB. Copyright © 2021 Hanna et al. 2021 Hanna et al. This content is distributed under the terms of the Creative Commons Attribution 4.0 International license.

We show here that, *in vitro*, M. marinum senses and reacts to elevated levels of Zn^2+^ by specifically inducing the expression of the Zn^2+^ efflux pump CtpC (Fig. 2B). Accordingly, growth of M. marinum Δ*ctpC* was inhibited at elevated concentrations of Zn^2+^ (Fig. 2C). Importantly, this growth inhibition was fully rescued by expressing the Mtb CtpC (Rv3270), confirming that CtpC (MMAR_1271) is its functional orthologue in Zn^2+^ efflux in M. marinum (Fig. 2C and D). Increasing concentrations of Mn^2+^ did not affect growth of M. marinum wt, nor that of the Δ*ctpC* mutant (Fig. S3F), excluding that M. marinum CtpC is a manganese exporter as was previously claimed for Mtb CtpC (56).

As shown for Mtb, M. marinum *ctpC* is also induced during infection of D. discoideum (Fig. 2A), and deletion of *ctpC* strongly attenuates intracellular growth of M. marinum (Fig. 2D). This indicates that, inside amoebae, M. marinum experiences toxic concentrations of Zn^2+^, which it resists by exporting Zn^2+^ through CtpC. Our results further demonstrate that MCV damage leads to the efflux of Zn^2+^ to the cytosol at early infection stages (Fig. 1), suggesting that the transient exposure to high Zn^2+^ levels is sufficient to stimulate *ctpC* expression. Therefore, it appears that M. marinum makes use of an integrated strategy to counteract the intoxication by Zn^2+^ in the MCV, first by increasing CtpC-mediated efflux into the MCV lumen and second by inducing ESAT-6-dependent MCV damage, resulting in leakage of ions (H^+^, Zn^2+^, and others) to the cytosol. This model is in line with a previous study (57) showing that accumulation of Zn^2+^ in Mtb-infected fibroblasts leads both to the upregulation of CtpC and to an increased secretion of ESAT-6 that results in phagosomal damage and ion efflux.

Strikingly, the intracellular growth of M. marinum wt was similarly impaired in amoebae lacking ZntA or ZntB (Fig. 5), although the levels of Zn^2+^ inside MCVs were higher in the D. discoideum *zntA* KO but lower in the *zntB* KO, compared to wt (Fig. 4). We propose the following model to account for this apparently counterintuitive result. First, the fact that an M. marinum mutant in a Zn-related gene (*ctpC*) is even more attenuated for intracellular growth in a D. discoideum mutant itself affected in a Zn-related gene (*zntA* or *zntB*) is a demonstration that the two mutants interact genetically in a way that is best explainable by a dual perturbation in Zn^2+^ homeostasis. Second, the increased attenuation of the Δ*ctpC* mutant in the *zntA* KO host is most likely due to the elevated MCV Zn^2+^ concentration, while the increased attenuation in the *zntB* KO is likely due to inefficient MCV escape and prolonged exposure to the residual lumenal Zn^2+^ concentration.

In summary, we conclude that D. discoideum restricts an infection with M. marinum by using one or more Zn^2+^-dependent host defense mechanisms, which are counteracted by the pathogen via the specific induction of its Zn^2+^ exporter CtpC. To our knowledge, this is the first study reporting the role of CtpC in the resistance of M. marinum to Zn^2+^-mediated host defense.

## MATERIALS AND METHODS

### D. discoideum strains, culture, and plasmids.

The D. discoideum material used in this study is listed in Table S3A in the supplemental material. The wt strains Ax2(Ka) and Ax4 were grown at 22°C in HL5-C medium (Formedium) supplemented with 100 U/ml penicillin and 100 μg/ml streptomycin. Overexpressers and KO cell lines were grown in medium with hygromycin (25 μg/ml), G418 (5 μg/ml), and/or blasticidin (5 μg/ml).

### *Mycobacterium* strains, culture, and plasmids.

The M. marinum strains and plasmids used in the present study are listed in Table S3B. M. marinum Δ*ctpC* was generated by specialized phage transduction as previously described (Table S3B) (19) with modifications. The flanking regions of *ctpC* were amplified using the primer pairs oHK143/oHK144 and oHK145/oHK146 (Table S3B). The PCR products were digested with AlwNI and Van91I, respectively, and ligated with Van91I-digested p0004S vector fragments. The resulting plasmid pHK42 and the DNA of the temperature-sensitive phage phAE159 were ligated after previous linearization with PacI, gaining pHK50. High-titer phages of pHK50 were prepared in M. smegmatis to perform specialized transduction in M. marinum, finally yielding the Δ*ctpC*::Hyg^r^ mutant. The phAE7.1 phage was used to remove the Hyg^r^ cassette (19). Primers used for mutant verification are listed in Table S3B (oHK159, oHK160, oHK33, and oHK36). A complementation vector was generated by the FX-cloning strategy (58) using the backbone pFLAG plasmid (59) to constitutively expresses the Mtb orthologue of *ctpC* (Rv3270) from the medium-strength tetracycline promoter. The vector is then stably integrated into the genomic *attB* site. Mtb *ctpC* was amplified from genomic DNA using the primer pair oHK375/oHK376 to yield pHK124. The correct insertion was verified by sequencing using the primers oHK328, oHK340, and oHK377. Mycobacteria were cultured at 150 rpm at 32°C in Middlebrook 7H9 (Difco) supplemented with 10% oleic acid-albumin-dextrose-catalase (OADC), 0.2% glycerol, and 0.05% Tween 80. Bacterial clumping was minimized by adding 5-mm glass beads during cultivation. Mutants and plasmid carriers were grown in medium supplemented with hygromycin (100 μg/ml), kanamycin (25 μg/ml), and/or ampicillin (100 μg/ml).

### M. marinum growth on agar and in broth.

M. marinum wt and Δ*ctpC* were grown in liquid 7H9-OADC-glycerol-Tween at 32°C in shaking until reaching an optical density at 600 nm (OD_600_) of approximately 0.5. For growth assays on agar, serial 10-fold dilutions were performed and 5 μl of each dilution was plated on 7H11-OADC-glycerol-Tween containing different concentrations (0, 0.125, 0.250, 0.500, 1.000, and 2.50 mM) of ZnSO_4_, MnCl_2_, and CuSO_4_. Pictures were taken after 7 days of incubation at 32°C. For growth in broth, overnight cultures of green fluorescent protein (GFP)-expressing M. marinum wt and Δ*ctpC* as well as of E2-Crimson (pTEC19, Addgene)-expressing wt and Δ*ctpC*::*ctpC* were diluted to 5 × 10^5^ bacteria/ml, centrifuged, and resuspended in 7H9 medium containing increasing ZnSO_4_ concentrations as indicated. Bacteria were distributed in 96-well plates at 10^5^/well (Perkin Elmer). Growth at 32°C under shaking conditions was assessed by measuring fluorescence at 509 nm for the GFP signal and 646 nm for the E2-Crimson signal in a plate reader (Synergy MX; BioTek). The growth of the Δ*ctpC* (GFP) and *ΔctpC*::*ctpC* (E2-Crimson) strains was normalized to the M. marinum wt strains expressing GFP and E2-Crimson, respectively.

### Infection of D. discoideum.

Infections were performed as previously described (21, 60). After spinoculation and washing off the extracellular bacteria, the infected cells were resuspended at a density of 1 × 10^6^ cells/ml in filtered HL5-C. Five micrograms per milliliter of streptomycin and 5 U/ml of penicillin were added to prevent extracellular bacterial growth. The infected cells were then incubated at 25°C at 130 rpm, and samples were taken for analysis at the indicated time points.

### Live cell imaging.

To monitor the subcellular localization of Zn^2+^ during infection, infected cells were plated on μ-dishes (ibidi), medium was exchanged to Soerensen buffer, and intracellular Zn^2+^ was stained with 5 μM NBD-TPEA (Sigma catalog no. N1040 [61]) for 30 min in the dark (33). To stain unlabeled bacteria, Vybrant DyeCycle Ruby stain (ThermoFisher) was used as previously described (3). Escape of M. marinum from MCVs was assessed in cells expressing mCherry-Plin. Images of live cells were taken with an inverted 3i Marianas spinning disc confocal microscope using the 63× glycerol or 100× oil objectives. Fluorescent proteins or probes were excited using the 488-nm (GFP and NBD-TPEA), 561-nm (mCherry), and 640-nm (Vybrant Ruby) laser lines, respectively. To quantify the integrated intensity of NBD-TPEA inside MCVs, cells were infected with mCherry-expressing M. marinum. At the indicated time points, samples were stained with NBD-TPEA and images were taken using the ImageXpress spinning disc confocal microscope (Molecular Devices). Here, mCherry-expressing bacteria were excited using the Texas Red laser line and NBD-TPEA-expressing bacteria were excited using the GFP laser line. The integrated intensity per MCV area was assessed using an analysis pipeline created in MetaXpress Custom Module Editor (Molecular Devices). Briefly, MCVs were defined as a sphere around mCherry-labeled bacteria that were detected using the Texas Red channel. NBD-TPEA inside MCVs was detected using the settings for the GFP channel. The normalized integrated intensity of NBD-TPEA was divided by the area of each MCV, leading to the integrated intensity per MCV area.

### Antibodies and immunofluorescence.

Antibodies against p80 (62) were from the Geneva Antibody Facility (University of Geneva, Switzerland); anti-Ub (FK2) monoclonal antibodies were from Enzo Life Sciences. The mCherry fluorescent signal was enhanced with rat monoclonal (MAb) anti-red fluorescent protein (anti-RFP) antibodies (Chromotek). As secondary antibodies, goat anti-rabbit, anti-mouse, and anti-rat IgG coupled to Alexa 488 or Alexa 546 (Thermo Fisher Scientific) or CF640R (Biotium) were used. Cells were fixed with cold methanol (MeOH) as described previously (40). Images were recorded with Zeiss LSM700 and LSM800 confocal microscopes and a 63×/1.4-numerical-aperture (NA) or a 100×/1.4-NA oil-immersion objective. Fluorescent proteins or secondary antibodies were excited using the 405-nm (DAPI), 488-nm (GFP and Alexa 488), 555-nm (Alexa 546), and 639-nm (CF640R) laser lines.

### RNA-sequencing data.

RNA-Seq data were gathered from the work of Hanna et al. (38). Briefly, infection was carried out using M. marinum expressing GFP as described above. In brief, at each time point, a homogeneous population of infected cells was obtained by GFP fluorescence-activated cell sorting (FACS). The total RNA was extracted, and the rRNA from host and bacteria was depleted, before generation of the cDNA libraries using a Bio-Rad iScript kit and sequencing. The resulting reads were mapped in parallel against the D. discoideum and M. marinum genome. Differential expression analysis of the infection time course was performed using the R package limma, by comparing on one hand infected D. discoideum cells to mock (i.e., buffer)-infected cells, and on the other hand intracellular M. marinum to M. marinum grown in broth.

### qRT-PCR sample collection and analysis.

To assess the effect of Zn^2+^ on the expression of *ctpC*, *ctpC*-like, *ctpV*, and *ctpV-*like, M. marinum strains were exposed to various concentrations of ZnSO_4_ (0.00, 0.05, 0.10, and 0.50 mM) for 2 h. Bacteria were harvested, RNA was extracted, and cDNA was synthesized using a Bio-Rad iScript kit. For each gene tested, the mean calculated threshold cycles (*C_T_*) were averaged and normalized to the *C_T_* of a gene with constant expression (*sigA*). The normalized *C_T_* was used for calculating the fold change using the ΔΔ*C_T_* method. Briefly, relative levels of target mRNA, normalized with respect to an endogenous control (*sigA*), were expressed as 2−ΔΔ*C_T_* (fold), where Δ*C_T_* = *C_T_* of the target gene − *C_T_* of the control gene (*sigA*), and ΔΔ*C_T_* = Δ*C_T_* of the studied set of conditions − Δ*C_T_* of the calibrator conditions, as previously described (63).

### Measurement of intracellular bacterial growth.

Intracellular growth of M. marinum expressing fluorescent reporters or bacterial luciferase was measured as previously described (64). Briefly, three different dilutions of infected cells (between 0.5 × 10^5^ and 2.0 × 10^5^ cells) were plated on nontreated, white F96 MicroWell plates (Nunc) or black 96-well plates (Perkin Elmer) covered with a gas-permeable moisture barrier seal (BioConcept). The course of infection was monitored by measuring either luminescence or fluorescence (at 509 and 646 nm) as a proxy of bacterial growth using a Synergy Mx microplate reader (BioTek) at a constant temperature of 25°C for around 70 h with 1-h intervals. The growth of the Δ*ctpC* (GFP) and *ΔctpC*::*ctpC* (E2-Crimson) strains was normalized to M. marinum wt strains expressing GFP and E2-Crimson, respectively.

### Phagocytic plaque assay.

Phagocytic plaque assays in the presence of nonpathogenic bacteria as food source were performed as described previously (43). Briefly, 50 μl of an overnight culture of various nonpathogenic bacteria was added to the wells of a 24-well plate containing 2 ml SM-agar. Then, 10, 100, 1,000, or 10,000 D. discoideum cells were added to the bacterial lawn and plates were incubated at 22°C for 4 to 7 days until plaques were visible. Quantification was performed by scoring the appearance of plaques in at least three independent experiments. The logarithmic plaquing score was defined as follows: plaque formation in wells with 10 amoebae yielded a score of 1,000; in the cases where cells did not grow at lower dilutions, they obtained the corresponding lower scores of 100, 10, and 1. The ability of D. discoideum Ax2(Ka), *zntA* KO, Ax4, and *zntB* KO to form plaques on M. marinum wt and Δ*ctpC* was assessed as described previously (43, 65). Briefly, 5 × 10^8^ mycobacteria were harvested and resuspended in 1.2 ml of 7H9-OADC-glycerol-Tween containing a 1:10^5^ dilution of an overnight culture of Klebsiella pneumoniae. Fifty microliters of the suspension was added to the wells of a 24-well plate containing 2 ml of 7H11-OADC-glycerol-Tween. Various dilutions of D. discoideum (i.e., 10, 100, 1,000, and 10,000 cells; for strains in the Ax4 background, 3-fold more cells were used to achieve similar temporal evolution of the plaques) were added to the bacterial lawn, and plates were incubated at 25°C for 7 days until plaques were visible. The scoring was performed as described above.

### Phylogenetic tree.

Putative P-type ATPases of M. marinum were identified by performing a protein BLAST search using NCBI PSI-BLAST and the sequence of Mtb CtpC. The results were then cross-compared to the annotations of M. marinum P-type ATPases available on Mycobrowser (66). The protein sequences of Mtb and M. marinum P-type ATPases were aligned with the online version of MAFFT (https://mafft.cbrc.jp/alignment/server/) using the G-INS-1 strategy and “leave gappy regions” to generate a phylogenetic tree in phylo.ilo using NJ conserved sites and the JTT substitution model (67).

### Data availability.

Information on the differential expression of M. marinum P-type ATPases (Table S1A) as well as D. discoideum CV genes (Table S1B), *zntA* to -*D* transporters (Table S1C), and *zplA* to -*G* transporters (Table S1D) was extracted from the preprint resource at https://doi.org/10.1101/590810 (38). Information on the enrichment of CV proteins at the MCV (Table S2) was extracted from the preprint resource at https://doi.org/10.1101/592717 (42).
