# Supplementary figures and images for: Zn2+ Intoxication of Mycobacterium marinum during Dictyostelium discoideum Infection Is Counteracted by Induction of the Pathogen Zn2+ Exporter CtpC
Source: mBio. 2021 Feb 2;12(1):e01313-20. doi: 10.1128/mBio.01313-20 (PMC7858047; doi:10.1128/mBio.01313-20)

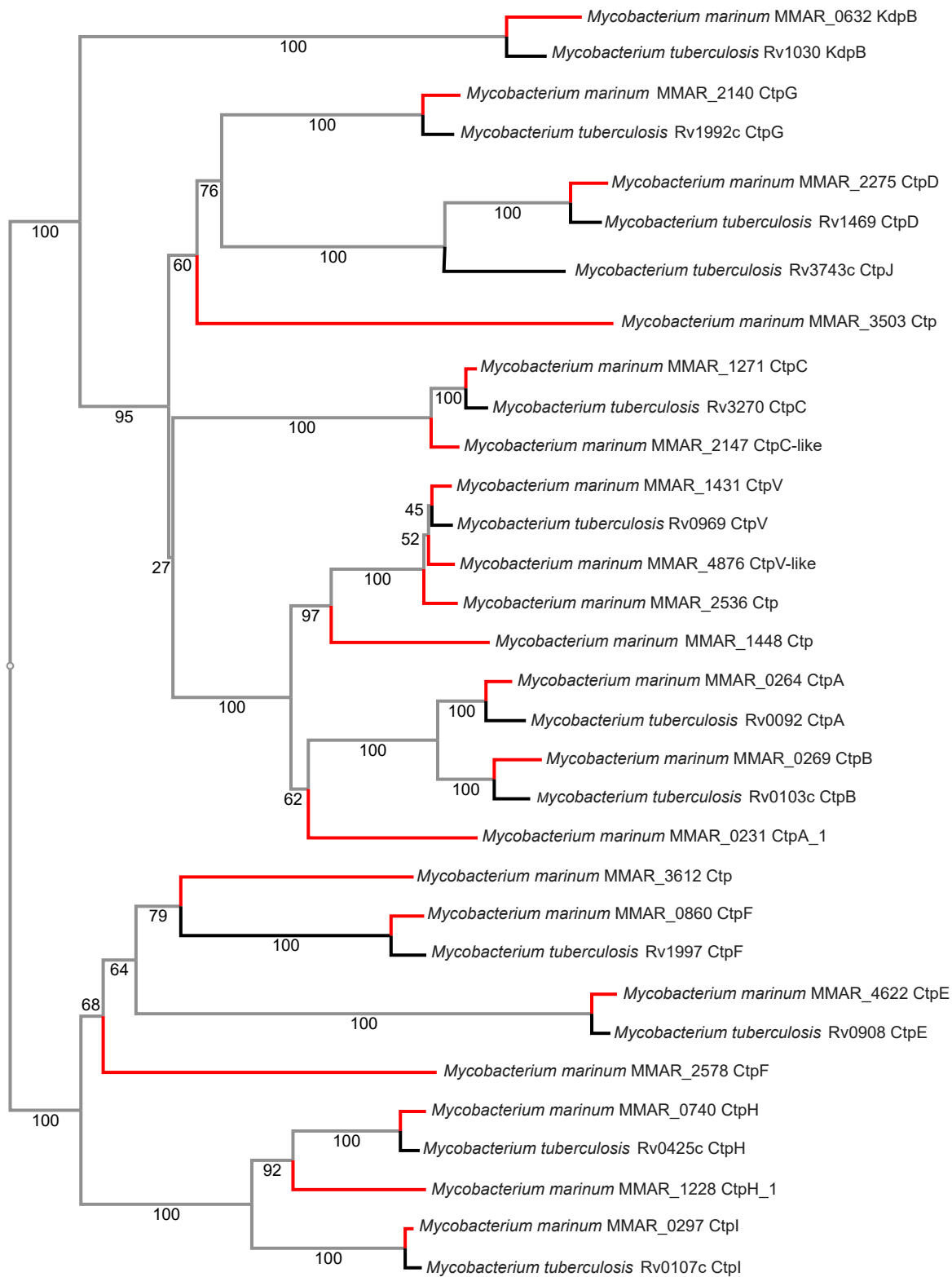

Supplement: FIG S1 [file mBio.01313-20-sf001.pdf]

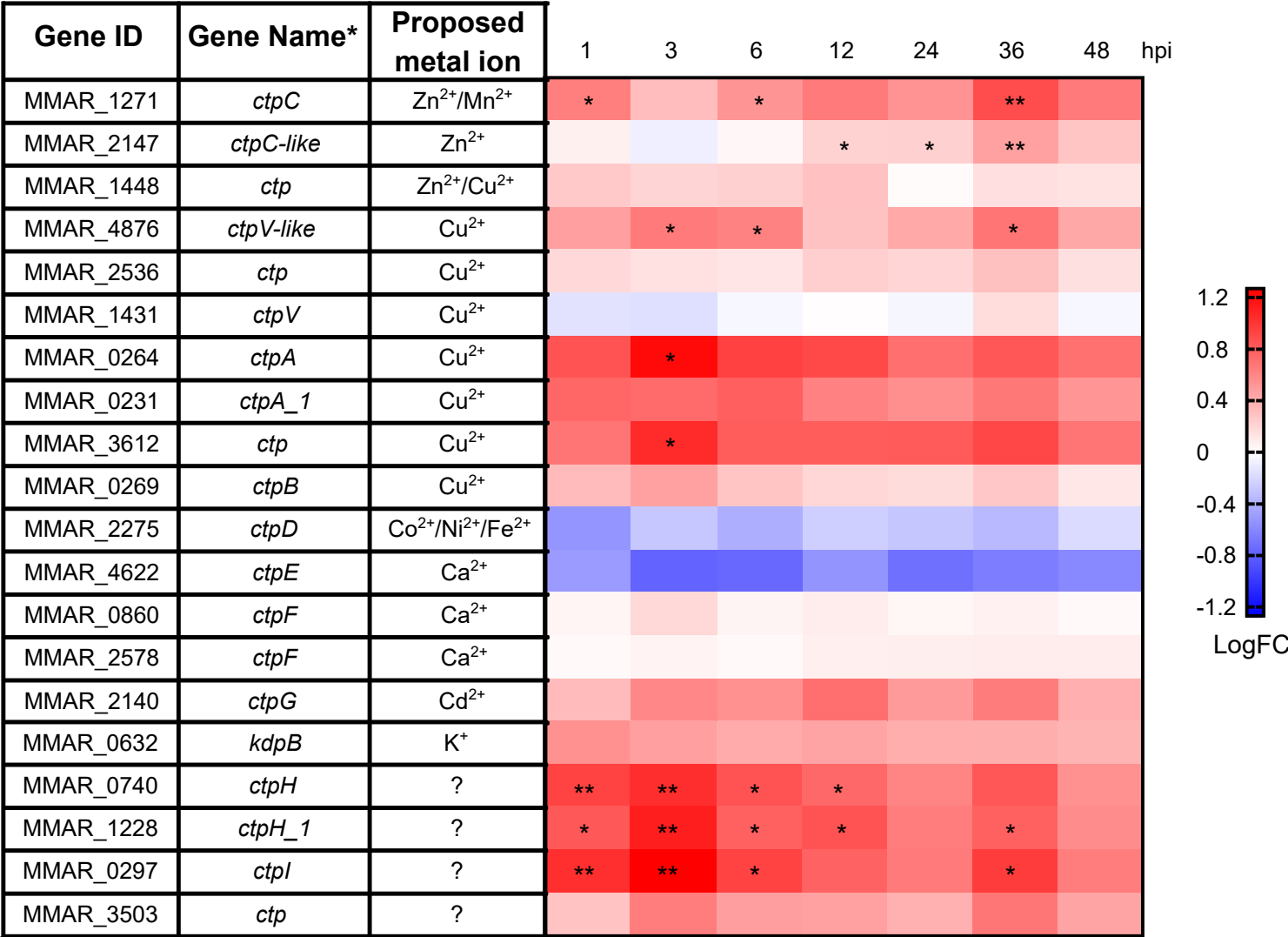

\* proposed gene name according to table S1

Supplement: FIG S2 [file mBio.01313-20-sf002.pdf]

Hanna *et al.*, Figure S3

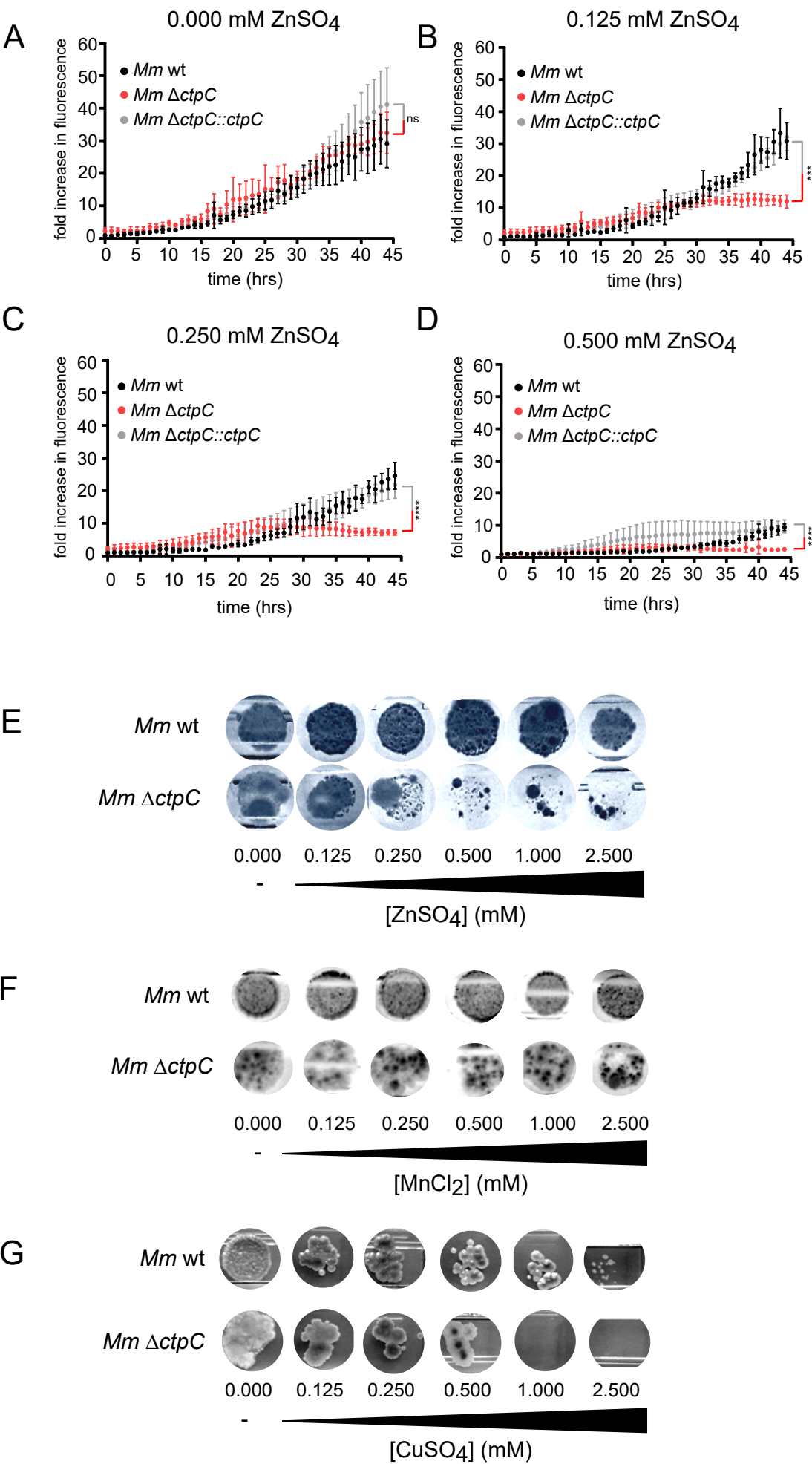

Supplement: FIG S3 [file mBio.01313-20-sf003.pdf]

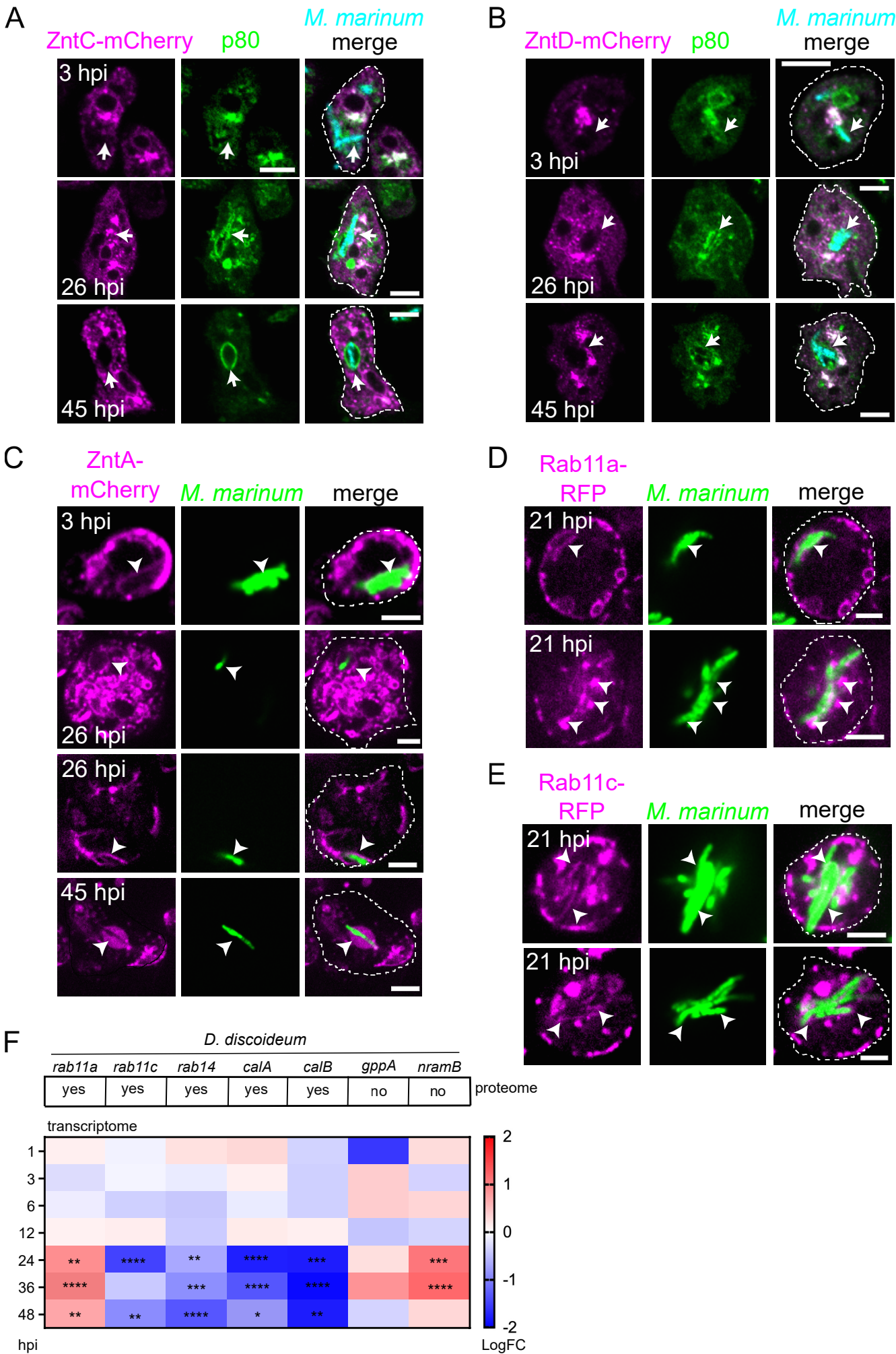

Supplement: FIG S4 [file mBio.01313-20-sf004.pdf]

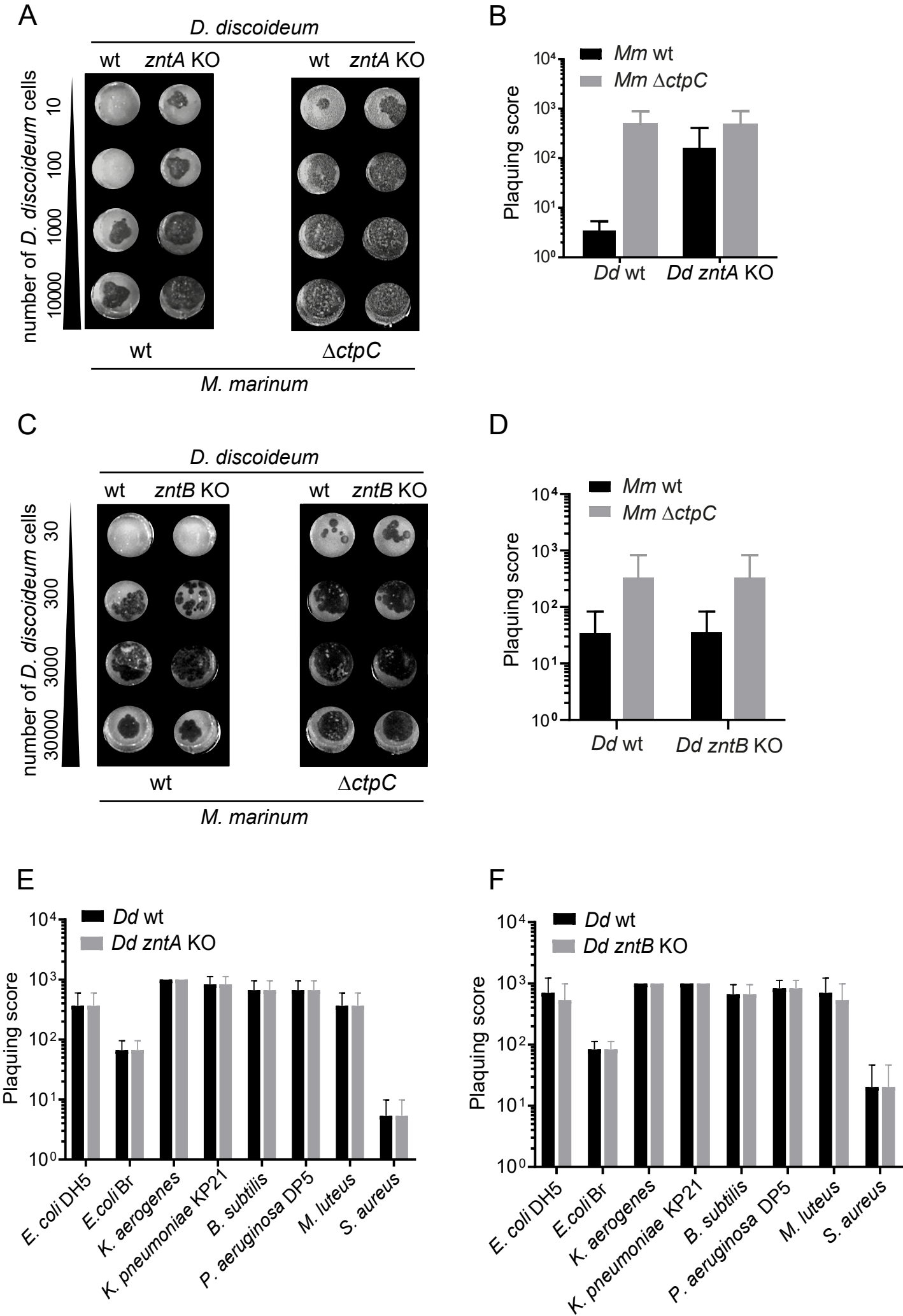

Supplement: FIG S5 [file mBio.01313-20-sf005.pdf]

A

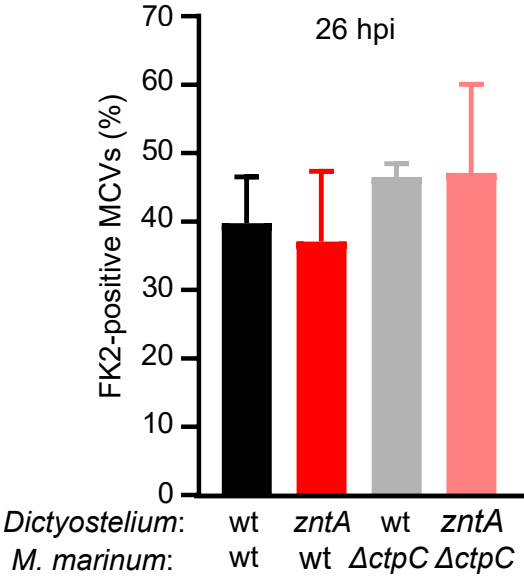

B

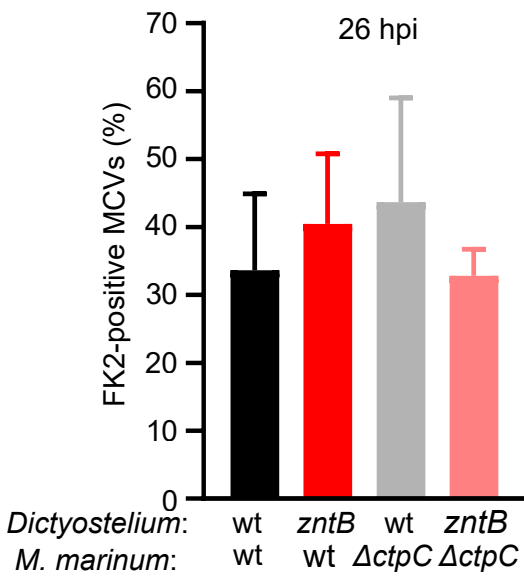

C

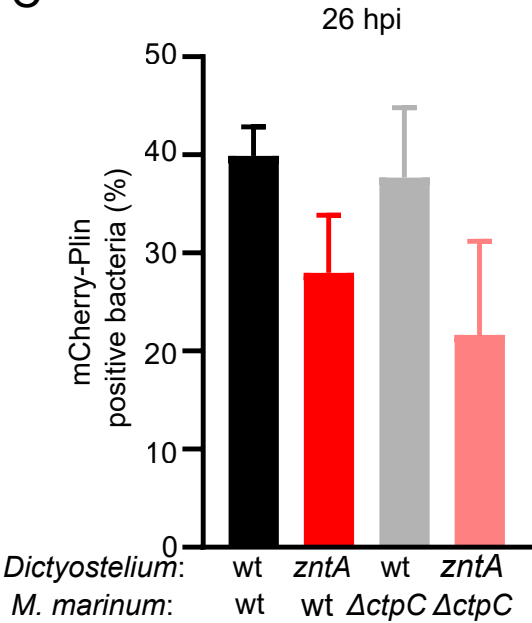

D

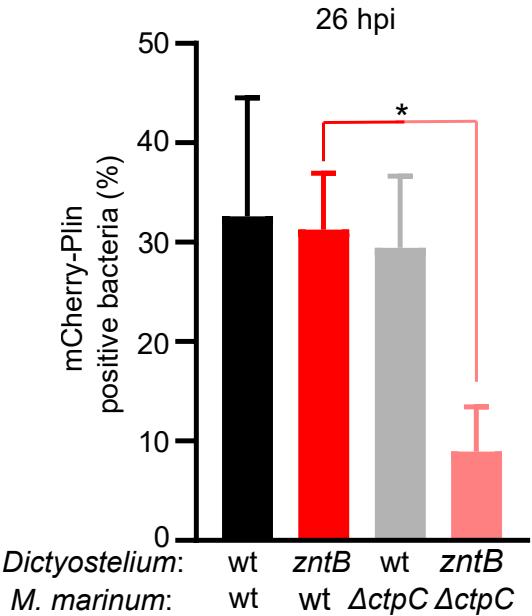

Supplement: FIG S6 [file mBio.01313-20-sf006.pdf]

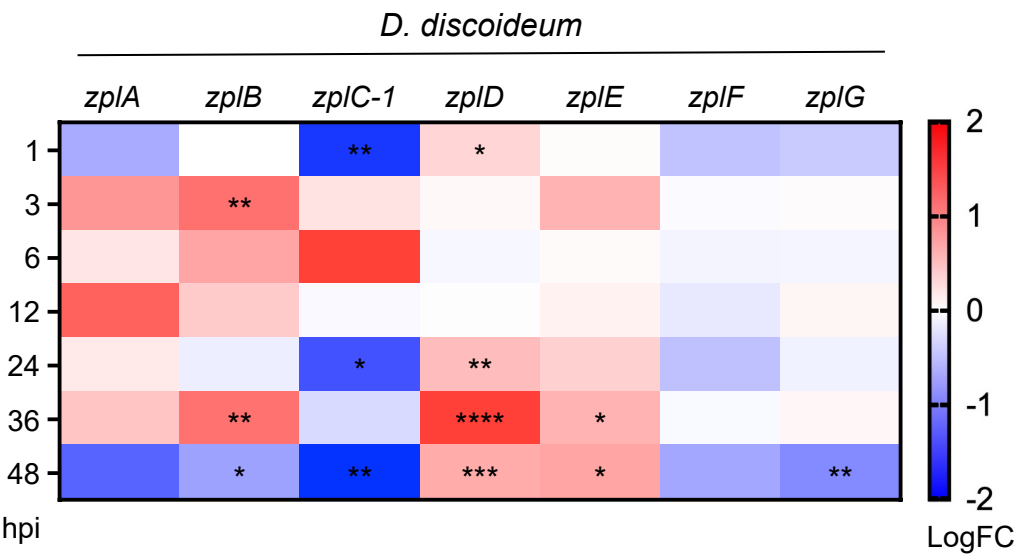

Supplement: FIG S7 [file mBio.01313-20-sf007.pdf]
