# Supplementary material for: Zn2+ Intoxication of Mycobacterium marinum during Dictyostelium discoideum Infection Is Counteracted by Induction of the Pathogen Zn2+ Exporter CtpC
Source: mBio. 2021 Feb 2;12(1):e01313-20. doi: 10.1128/mBio.01313-20 (PMC7858047; doi:10.1128/mBio.01313-20)
Supplement: TABLE S1 [file mBio.01313-20-st001.docx]

| **S1A Table. Differential expression of *M. marinum* p-type ATPase genes during infection** | | | | | | | | | |  |
| --- | --- | --- | --- | --- | --- | --- | --- | --- | --- | --- |
| **Gene ID** | **Product** | | **Proposed gene Name*** | | **LogFC** | | **Adj. *p* value** | | **Time point (hpi)** |  |
| MMAR_1271 | Metal cation-transporting p-type ATPase C CtpC | | *ctpC* | | 0.641 | | 0.022 | | 1 |  |
|  |  |  |  |  | 0.325 | | 0.257 | | 3 |  |
|  |  |  |  |  | 0.529 | | 0.049 | | 6 |  |
|  |  |  |  |  | 0.659 | | 0.051 | | 12 |  |
|  |  |  |  |  | 0.533 | | 0.132 | | 24 |  |
|  |  |  |  |  | 0.888 | | 0.005 | | 36 |  |
|  |  |  |  |  | 0.657 | | 0.104 | | 48 |  |
| MMAR_2145 | Conserved hypothetical protein | | *ctpC-like* | | 0.071 | | 0.716 | | 1 |  |
|  |  |  |  |  | -0.080 | | 0.707 | | 3 |  |
|  |  |  |  |  | 0.035 | | 0.849 | | 6 |  |
|  |  |  |  |  | 0.220 | | 0.250 | | 12 |  |
|  |  |  |  |  | 0.231 | | 0.224 | | 24 |  |
|  |  |  |  |  | 0.464 | | 0.023 | | 36 |  |
|  |  |  |  |  | 0.288 | | 0.116 | | 48 |  |
| MMAR_1431 | Cation transport ATPase, ZntA | | *ctpV* | | -0.148 | | 0.497 | | 1 |  |
|  |  |  |  |  | -0.164 | | 0.464 | | 3 |  |
|  |  |  |  |  | -0.041 | | 0.855 | | 6 |  |
|  |  |  |  |  | 0.006 | | 0.982 | | 12 |  |
|  |  |  |  |  | -0.050 | | 0.869 | | 24 |  |
|  |  |  |  |  | 0.164 | | 0.453 | | 36 |  |
|  |  |  |  |  | -0.043 | | 0.889 | | 48 |  |
| MMAR_4876 | Metal cation transporter p-type ATPase, CtpV | | *ctpV-like1* | | 0.474 | | 0.136 | | 1 |  |
|  |  |  |  |  | 0.653 | | 0.048 | | 3 |  |
|  |  |  |  |  | 0.618 | | 0.049 | | 6 |  |
|  |  |  |  |  | 0.300 | | 0.386 | | 12 |  |
|  |  |  |  |  | 0.425 | | 0.267 | | 24 |  |
|  |  |  |  |  | 0.686 | | 0.049 | | 36 |  |
|  |  |  |  |  | 0.430 | | 0.286 | | 48 |  |
| MMAR_2536 | Metal cation transporter p-type ATPase | | *ctp* | | 0.186 | | 0.541 | | 1 |  |
|  |  |  |  |  | 0.145 | | 0.654 | | 3 |  |
|  |  |  |  |  | 0.128 | | 0.673 | | 6 |  |
|  |  |  |  |  | 0.237 | | 0.456 | | 12 |  |
|  |  |  |  |  | 0.207 | | 0.548 | | 24 |  |
|  |  |  |  |  | 0.310 | | 0.294 | | 36 |  |
|  |  |  |  |  | 0.150 | | 0.693 | | 48 |  |
| MMAR_1448 | Zinc cation transport ATPase | | *ctp* | | 0.268 | | 0.287 | | 1 |  |
|  |  |  |  |  | 0.214 | | 0.431 | | 3 |  |
|  |  |  |  |  | 0.230 | | 0.348 | | 6 |  |
|  |  |  |  |  | 0.308 | | 0.264 | | 12 |  |
|  |  |  |  |  | 0.017 | | 0.966 | | 24 |  |
|  |  |  |  |  | 0.155 | | 0.573 | | 36 |  |
|  |  |  |  |  | 0.135 | | 0.681 | | 48 |  |
| MMAR_0264 | Cation transporter p-type ATPase a CtpA | | *ctpA* | | 0.854 | | 0.097 | | 1 |  |
|  |  |  |  |  | 1.221 | | 0.027 | | 3 |  |
|  |  |  |  |  | 0.943 | | 0.060 | | 6 |  |
|  |  |  |  |  | 0.904 | | 0.114 | | 12 |  |
|  |  |  |  |  | 0.713 | | 0.251 | | 24 |  |
|  |  |  |  |  | 0.835 | | 0.113 | | 36 |  |
|  |  |  |  |  | 0.707 | | 0.283 | | 48 |  |
| MMAR_0231 | Cation transporter p-type ATPase CtpA_1 | | *ctpA_1* | | 0.761 | | 0.081 | | 1 |  |
|  |  |  |  |  | 0.739 | | 0.095 | | 3 |  |
|  |  |  |  |  | 0.792 | | 0.062 | | 6 |  |
|  |  |  |  |  | 0.626 | | 0.183 | | 12 |  |
|  |  |  |  |  | 0.552 | | 0.288 | | 24 |  |
|  |  |  |  |  | 0.670 | | 0.131 | | 36 |  |
|  |  |  |  |  | 0.525 | | 0.329 | | 48 |  |
| MMAR_3612 | Metal cation transporter p-type ATPase a | | *ctp* | | 0.686 | | 0.168 | | 1 |  |
|  |  |  |  |  | 1.055 | | 0.043 | | 3 |  |
|  |  |  |  |  | 0.808 | | 0.093 | | 6 |  |
|  |  |  |  |  | 0.808 | | 0.147 | | 12 |  |
|  |  |  |  |  | 0.808 | | 0.147 | | 24 |  |
|  |  |  |  |  | 0.812 | | 0.189 | | 36 |  |
|  |  |  |  |  | 0.913 | | 0.079 | | 48 |  |
| MMAR_0269 | Cation-transporter p-type ATPase B CtpB | | *ctpB* | | 0.337 | | 0.190 | | 1 |  |
|  |  |  |  |  | 0.466 | | 0.084 | | 3 |  |
|  |  |  |  |  | 0.284 | | 0.252 | | 6 |  |
|  |  |  |  |  | 0.198 | | 0.492 | | 12 |  |
|  |  |  |  |  | 0.173 | | 0.581 | | 24 |  |
|  |  |  |  |  | 0.278 | | 0.298 | | 36 |  |
|  |  |  |  |  | 0.117 | | 0.738 | | 48 |  |
| MMAR_2275 | Cation transporter p-type ATPase D CtpD | | *ctpD* | | -0.532 | | 0.066 | | 1 |  |
|  |  |  |  |  | -0.282 | | 0.346 | | 3 |  |
|  |  |  |  |  | -0.406 | | 0.134 | | 6 |  |
|  |  |  |  |  | -0.244 | | 0.432 | | 12 |  |
|  |  |  |  |  | -0.287 | | 0.390 | | 24 |  |
|  |  |  |  |  | -0.353 | | 0.224 | | 36 |  |
|  |  |  |  |  | -0.184 | | 0.611 | | 48 |  |
| MMAR_4622 | Metal cation transporter ATPase p-type CtpE | | *ctpE* | | -0.507 | | 0.137 | | 1 |  |
|  |  |  |  |  | -0.780 | | 0.036 | | 3 |  |
|  |  |  |  |  | -0.757 | | 0.029 | | 6 |  |
|  |  |  |  |  | -0.535 | | 0.158 | | 12 |  |
|  |  |  |  |  | -0.715 | | 0.127 | | 24 |  |
|  |  |  |  |  | -0.650 | | 0.073 | | 36 |  |
|  |  |  |  |  | -0.595 | | 0.217 | | 48 |  |
| MMAR_0860 | Metal cation-transporting p-type ATPase F, CtpF | | *ctpF* | | 0.048 | | 0.826 | | 1 |  |
|  |  |  |  |  | 0.192 | | 0.320 | | 3 |  |
|  |  |  |  |  | 0.053 | | 0.791 | | 6 |  |
|  |  |  |  |  | 0.085 | | 0.691 | | 12 |  |
|  |  |  |  |  | 0.040 | | 0.882 | | 24 |  |
|  |  |  |  |  | 0.065 | | 0.764 | | 36 |  |
|  |  |  |  |  | 0.029 | | 0.917 | | 48 |  |
| MMAR_2578 | Metal cation transporter p-type ATPase a, CtpF | | *ctpF* | | 0.029 | | 0.938 | | 1 |  |
|  |  |  |  |  | 0.057 | | 0.869 | | 3 |  |
|  |  |  |  |  | 0.028 | | 0.931 | | 6 |  |
|  |  |  |  |  | 0.077 | | 0.820 | | 12 |  |
|  |  |  |  |  | 0.083 | | 0.831 | | 24 |  |
|  |  |  |  |  | 0.092 | | 0.779 | | 36 |  |
|  |  |  |  |  | 0.094 | | 0.804 | | 48 |  |
| MMAR_2140 | Metal cation transporter p-type ATPase | | *ctpG* | | 0.334 | | 0.409 | | 1 |  |
|  |  |  |  |  | 0.592 | | 0.134 | | 3 |  |
|  |  |  |  |  | 0.545 | | 0.138 | | 6 |  |
|  |  |  |  |  | 0.711 | | 0.103 | | 12 |  |
|  |  |  |  |  | 0.500 | | 0.284 | | 24 |  |
|  |  |  |  |  | 0.649 | | 0.105 | | 36 |  |
|  |  |  |  |  | 0.395 | | 0.408 | | 48 |  |
| MMAR_0740 | Metal cation transporting p-type ATPase CtpH | | *ctpH* | | 0.935 | | 0.007 | | 1 |  |
|  |  |  |  |  | 1.037 | | 0.009 | | 3 |  |
|  |  |  |  |  | 0.853 | | 0.015 | | 6 |  |
|  |  |  |  |  | 0.747 | | 0.071 | | 12 |  |
|  |  |  |  |  | 0.612 | | 0.157 | | 24 |  |
|  |  |  |  |  | 0.833 | | 0.029 | | 36 |  |
|  |  |  |  |  | 0.543 | | 0.233 | | 48 |  |
| MMAR_1228 | Metal cation transporting p-type ATPase CtpH_1 | | *ctpH_1* | | 0.830 | | 0.021 | | 1 |  |
|  |  |  |  |  | 1.116 | | 0.007 | | 3 |  |
|  |  |  |  |  | 0.785 | | 0.026 | | 6 |  |
|  |  |  |  |  | 0.852 | | 0.051 | | 12 |  |
|  |  |  |  |  | 0.646 | | 0.153 | | 24 |  |
|  |  |  |  |  | 0.790 | | 0.043 | | 36 |  |
|  |  |  |  |  | 0.572 | | 0.233 | | 48 |  |
| MMAR_0297 | Cation-transporter ATPase I CtpI | | *ctpI* | | 1.030 | | 0.007 | | 1 |  |
|  |  |  |  |  | 1.268 | | 0.005 | | 3 |  |
|  |  |  |  |  | 0.936 | | 0.015 | | 6 |  |
|  |  |  |  |  | 0.777 | | 0.081 | | 12 |  |
|  |  |  |  |  | 0.660 | | 0.165 | | 24 |  |
|  |  |  |  |  | 0.966 | | 0.024 | | 36 |  |
|  |  |  |  |  | 0.646 | | 0.217 | | 48 |  |
| MMAR_0632 | High-affinity K+ transport system, ATPase chain B, KdpB | | *kdpB* | | 0.542 | | 0.052 | | 1 |  |
|  |  |  |  |  | 0.473 | | 0.098 | | 3 |  |
|  |  |  |  |  | 0.415 | | 0.115 | | 6 |  |
|  |  |  |  |  | 0.449 | | 0.143 | | 12 |  |
|  |  |  |  |  | 0.404 | | 0.227 | | 24 |  |
|  |  |  |  |  | 0.402 | | 0.159 | | 36 |  |
|  |  |  |  |  | 0.371 | | 0.293 | | 48 |  |
| MMAR_3503 | Metal cation transporter p-type ATPase | | *ctp* | | 0.294 | | 0.444 | | 1 |  |
|  |  |  |  |  | 0.646 | | 0.087 | | 3 |  |
|  |  |  |  |  | 0.481 | | 0.166 | | 6 |  |
|  |  |  |  |  | 0.466 | | 0.247 | | 12 |  |
|  |  |  |  |  | 0.391 | | 0.367 | | 24 |  |
|  |  |  |  |  | 0.678 | | 0.079 | | 36 |  |
|  |  |  |  |  | 0.448 | | 0.328 | | 48 |  |
| * The proposed gene names are based on the information gathered from https://mycobrowser.epfl.ch/ website based on the homology with *Mycobacterium tuberculosis* | | | | | | | | | |  |
|  |  |  |  |  |  |  |  |  |  |  |
| **S1B Table. Differential expression of *D. discoideum* CV genes during**  ***M. marinum* infection.** | | | | | | | | | | |
| **Gene ID** | | **Gene name** | | **LogFC** | | **Adjusted *p* value** | | **Timepoint (hpi)** | | |
| DDB_G0269238 | | *rab11a* | | 0.079 | | 0.781 | | 1 | | |
|  |  |  |  | -0.251 | | 0.862 | | 3 | | |
|  |  |  |  | -0.045 | | 0.979 | | 6 | | |
|  |  |  |  | 0.065 | | 0.912 | | 12 | | |
|  |  |  |  | 0.961 | | 0.001 | | 24 | | |
|  |  |  |  | 1.173 | | 1.89E-05 | | 36 | | |
|  |  |  |  | 0.766 | | 0.006 | | 48 | | |
| DDB_G0277101 | | *rab11c* | | -0.041 | | 0.916 | | 1 | | |
|  |  |  |  | -0.034 | | 0.993 | | 3 | | |
|  |  |  |  | -0.227 | | 0.879 | | 6 | | |
|  |  |  |  | 0.133 | | 0.862 | | 12 | | |
|  |  |  |  | -1.442 | | 1.78E-05 | | 24 | | |
|  |  |  |  | -0.526 | | 0.125 | | 36 | | |
|  |  |  |  | -0.964 | | 0.003 | | 48 | | |
| DDB_G0281337 | | *rab14* | | 0.193 | | 0.270 | | 1 | | |
|  |  |  |  | -0.064 | | 0.781 | | 3 | | |
|  |  |  |  | -0.182 | | 0.488 | | 6 | | |
|  |  |  |  | -0.284 | | 0.191 | | 12 | | |
|  |  |  |  | -0.786 | | 0.002 | | 24 | | |
|  |  |  |  | -0.920 | | 0.000 | | 36 | | |
|  |  |  |  | -1.358 | | 1.08E-07 | | 48 | | |
| DDB_G0279407 | | *calA* | | 0.203 | | 0.625 | | 1 | | |
|  |  |  |  | 0.074 | | 0.980 | | 3 | | |
|  |  |  |  | -0.092 | | 0.972 | | 6 | | |
|  |  |  |  | 0.185 | | 0.832 | | 12 | | |
|  |  |  |  | -1.825 | | 4.60E-05 | | 24 | | |
|  |  |  |  | -1.404 | | 0.001 | | 36 | | |
|  |  |  |  | -0.894 | | 0.040 | | 48 | | |
| DDB_G0269104 | | *calB* | | -0.201 | | 0.709 | | 1 | | |
|  |  |  |  | -0.319 | | 0.930 | | 3 | | |
|  |  |  |  | -0.228 | | 0.935 | | 6 | | |
|  |  |  |  | 0.168 | | 0.887 | | 12 | | |
|  |  |  |  | -1.849 | | 0.000 | | 24 | | |
|  |  |  |  | -2.156 | | 1.97E-05 | | 36 | | |
|  |  |  |  | -1.753 | | 0.000 | | 48 | | |
| DDB_G0267398 | | *gppA* | | -1.582 | | 0.024 | | 1 | | |
|  |  |  |  | 0.529 | | 0.929 | | 3 | | |
|  |  |  |  | 0.478 | | 0.891 | | 6 | | |
|  |  |  |  | -0.368 | | 0.808 | | 12 | | |
|  |  |  |  | 0.277 | | 0.731 | | 24 | | |
|  |  |  |  | 0.967 | | 0.218 | | 36 | | |
|  |  |  |  | -0.232 | | 0.748 | | 48 | | |
| DDB_G0275815 | | *nrampB* | | 0.304 | | 0.294 | | 1 | | |
|  |  |  |  | -0.226 | | 0.926 | | 3 | | |
|  |  |  |  | 0.346 | | 0.772 | | 6 | | |
|  |  |  |  | -0.204 | | 0.742 | | 12 | | |
|  |  |  |  | 1.107 | | 0.000 | | 24 | | |
|  |  |  |  | 1.250 | | 6.88E-05 | | 36 | | |
|  |  |  |  | 0.416 | | 0.172 | | 48 | | |
|  | |  | |  | |  | |  | | |
| **S1C Table. Differential expression of *D. discoideum* *zntA-D* transporters during *M. marinum* infection.** | | | | | | | | | | |
| **Gene ID** | | **Gene name** | | **LogFC** | | **Adjusted *p* value** | | **Time point (hpi)** | | |
| DDB_G0283629 | | *zntA* | | 0.7376 | | 4.86E-05 | | 1 | | |
|  |  |  |  | -0.0593 | | 0.969 | | 3 | | |
|  |  |  |  | 0.4146 | | 0.470 | | 6 | | |
|  |  |  |  | 0.3508 | | 0.304 | | 12 | | |
|  |  |  |  | 1.2701 | | 3.70E-08 | | 24 | | |
|  |  |  |  | 1.2005 | | 1.55E-07 | | 36 | | |
|  |  |  |  | 1.8154 | | 1.41E-13 | | 48 | | |
| DDB_G0282067 | | *zntB* | | 2.74 | | 2.73E-23 | | 1 | | |
|  |  |  |  | 0.8484 | | 0.114 | | 3 | | |
|  |  |  |  | 0.7464 | | 0.213 | | 6 | | |
|  |  |  |  | 0.3817 | | 0.426 | | 12 | | |
|  |  |  |  | 1.2188 | | 0.000 | | 24 | | |
|  |  |  |  | 0.664 | | 0.040 | | 36 | | |
|  |  |  |  | 1.0877 | | 0.001 | | 48 | | |
| DDB_G0269332 | | *zntC* | | 0.1333 | | 0.589 | | 1 | | |
|  |  |  |  | -0.0942 | | 0.952 | | 3 | | |
|  |  |  |  | 0.054 | | 0.972 | | 6 | | |
|  |  |  |  | -0.457 | | 0.201 | | 12 | | |
|  |  |  |  | -0.5898 | | 0.026 | | 24 | | |
|  |  |  |  | -0.9956 | | 0.000 | | 36 | | |
|  |  |  |  | -0.501 | | 0.052 | | 48 | | |
| DDB_G0291141 | | *zntD* | | -0.8446 | | 0.001 | | 1 | | |
|  |  |  |  | 0.2956 | | 0.877 | | 3 | | |
|  |  |  |  | 0.208 | | 0.872 | | 6 | | |
|  |  |  |  | 0.1851 | | 0.755 | | 12 | | |
|  |  |  |  | -0.3203 | | 0.318 | | 24 | | |
|  |  |  |  | 0.2661 | | 0.440 | | 36 | | |
|  |  |  |  | -0.7934 | | 0.007 | | 48 | | |
|  | |  | |  | |  | |  | | |
| **S1DTable. Differential expression of *D. discoideum* *zplA-G* transporters during *M. marinum* infection** | | | | | | | | | | |
| **Gene ID** | | **Gene name** | | **LogFC** | | **Adjusted *p* value** | | **Time point (hpi)** | | |
| DDB_G0281921 | | *zplA* | | -0.8195 | | 0.2616 | | 1 | | |
|  |  |  |  | 0.9873 | | 0.3503 | | 3 | | |
|  |  |  |  | 0.2535 | | 0.8261 | | 6 | | |
|  |  |  |  | 1.4384 | | 0.1338 | | 12 | | |
|  |  |  |  | 0.2091 | | 0.8201 | | 24 | | |
|  |  |  |  | 0.5731 | | 0.5533 | | 36 | | |
|  |  |  |  | -1.4608 | | 0.0819 | | 48 | | |
| DDB_G0271672 | | *zplB* | | -0.0077 | | 0.9821 | | 1 | | |
|  |  |  |  | 1.3115 | | 0.0087 | | 3 | | |
|  |  |  |  | 0.8543 | | 0.0538 | | 6 | | |
|  |  |  |  | 0.5082 | | 0.2246 | | 12 | | |
|  |  |  |  | -0.178 | | 0.683 | | 24 | | |
|  |  |  |  | 1.2943 | | 0.0048 | | 36 | | |
|  |  |  |  | -0.906 | | 0.0357 | | 48 | | |
| DDB_G0273091 | | *zplC-1* | | -1.8767 | | 0.0033 | | 1 | | |
|  |  |  |  | 0.276 | | 0.7688 | | 3 | | |
|  |  |  |  | 1.6964 | | 0.0823 | | 6 | | |
|  |  |  |  | -0.069 | | 0.9365 | | 12 | | |
|  |  |  |  | -1.6164 | | 0.0274 | | 24 | | |
|  |  |  |  | -0.3702 | | 0.6356 | | 36 | | |
|  |  |  |  | -2.0029 | | 0.0044 | | 48 | | |
| DDB_G0269326 | | *zplD* | | 0.4059 | | 0.0249 | | 1 | | |
|  |  |  |  | 0.0641 | | 0.8081 | | 3 | | |
|  |  |  |  | -0.0913 | | 0.7256 | | 6 | | |
|  |  |  |  | 0.013 | | 0.954 | | 12 | | |
|  |  |  |  | 0.6496 | | 0.0064 | | 24 | | |
|  |  |  |  | 1.7024 | | 1.97E-11 | | 36 | | |
|  |  |  |  | 0.8004 | | 0.0006 | | 48 | | |
| DDB_G0286049 | | *zplE* | | 0.0328 | | 0.904 | | 1 | | |
|  |  |  |  | 0.7273 | | 0.1376 | | 3 | | |
|  |  |  |  | 0.0499 | | 0.9176 | | 6 | | |
|  |  |  |  | 0.1289 | | 0.7419 | | 12 | | |
|  |  |  |  | 0.4596 | | 0.1983 | | 24 | | |
|  |  |  |  | 0.7309 | | 0.0411 | | 36 | | |
|  |  |  |  | 0.0411 | | 0.014 | | 48 | | |
| DDB_G0286345 | | *zplF* | | -0.604 | | 0.1322 | | 1 | | |
|  |  |  |  | -0.045 | | 0.9356 | | 3 | | |
|  |  |  |  | -0.1124 | | 0.8299 | | 6 | | |
|  |  |  |  | -0.233 | | 0.5898 | | 12 | | |
|  |  |  |  | -0.6165 | | 0.1832 | | 24 | | |
|  |  |  |  | -0.0583 | | 0.9002 | | 36 | | |
|  |  |  |  | -0.8588 | | 0.0559 | | 48 | | |
| DDB_G0268426 | | *zplG* | | -0.5226 | | 0.09 | | 1 | | |
|  |  |  |  | 0.0318 | | 0.9479 | | 3 | | |
|  |  |  |  | -0.1086 | | 0.8117 | | 6 | | |
|  |  |  |  | 0.1023 | | 0.7941 | | 12 | | |
|  |  |  |  | -0.1404 | | 0.7148 | | 24 | | |
|  |  |  |  | 0.0796 | | 0.8283 | | 36 | | |
|  |  |  |  | -1.1228 | | 0.0024 | | 48 | | |
