# Supplementary material for: Zn2+ Intoxication of Mycobacterium marinum during Dictyostelium discoideum Infection Is Counteracted by Induction of the Pathogen Zn2+ Exporter CtpC
Source: mBio. 2021 Feb 2;12(1):e01313-20. doi: 10.1128/mBio.01313-20 (PMC7858047; doi:10.1128/mBio.01313-20)
Supplement: TABLE S2 [file mBio.01313-20-st002.docx]

| **S2 Table. Enrichment of CV proteins at the MCV** | | |
| --- | --- | --- |
| **Gene ID** | **Gene name** | **MCV** |
| DDB_G0268632 | *abpA* | yes |
| DDB_G0278495 | *alp* | yes |
| DDB_G0273439 | *ap2a1-1* | no |
| DDB_G0285793 | *cadA* | yes |
| DDB_G0279407 | *calA* | yes |
| DDB_G0269104 | *calB* | yes |
| DDB_G0278685 | *clmA* | yes |
| DDB_G0291157 | *cln3* | no |
| DDB_G0280955 | *dsgg* | no |
| DDB_G0277851 | *dymB* | no |
| DDB_G0291512 | *epnA* | no |
| DDB_G0287271 | *gol* | no |
| DDB_G0267398 | *gppA* | no |
| DDB_G0285703 | *hipA* | no |
| DDB_G0274805 | *ibrA* | yes |
| DDB_G0269150 | *lvsA* | yes |
| DDB_G0271502 | *lvsD* | no |
| DDB_G0290233 | *mgp1* | no |
| DDB_G0270024 | *mgp2* | yes |
| DDB_G0269624 | *mgp3* | no |
| DDB_G0283899 | *mgp4* | no |
| DDB_G0272112 | *myoJ* | yes |
| DDB_G0275815 | *nrampB* | no |
| DDB_G0269130 | *phgA* | no |
| DDB_G0269238 | *rab11A* | yes |
| DDB_G0277101 | *rab11C* | yes |
| DDB_G0281337 | *rab14* | yes |
| DDB_G0280043 | *rab8A* | yes |
| DDB_G0269240 | *racH* | yes |
| DDB_G0283389 | *rhgA* | yes |
| DDB_G0270986 | *tspC* | no |
| DDB_G0277173 | *vamp7B* | yes |
| DDB_G0287127 | *vatA* | yes |
| DDB_G0291858 | *vatM* | yes |
| DDB_G0285175 | *vmp1* | no |
| DDB_G0292974 | *vti1A* | yes |
| DDB_G0268144 | *vwkA* | yes |
| Analysis of recent proteomic data of early *M. marinum* MCVs (Table S1 of [42]) reveals a possible crosstalk between the CV and the MCV. 37 proteins annotated on dictybase with the GO term “Contractile Vacuole” were analysed for their presence in the MCV proteome. For more information, please see Guého et al. [42]. | | |
