# Supplementary material for: Zn2+ Intoxication of Mycobacterium marinum during Dictyostelium discoideum Infection Is Counteracted by Induction of the Pathogen Zn2+ Exporter CtpC
Source: mBio. 2021 Feb 2;12(1):e01313-20. doi: 10.1128/mBio.01313-20 (PMC7858047; doi:10.1128/mBio.01313-20)
Supplement: TABLE S3 [file mBio.01313-20-st003.docx]

| **S3A Table. *D. discoideum* material used for this study.** | | |
| --- | --- | --- |
| **Strains** | **Relevant characteristics** | **Source/Reference** |
| Ax2(Ka) | wt, parental strain of the *zntA* KO |  |
| Ax2(Ka) *zntA* KO | BSD^r^ | [33] |
| AX4 | wt, parental strain of the *zntB* KO |  |
| AX4 *zntB* KO | BSD^r^ | REMI library, Prof. Christopher Thompson [33] |
|  |  |  |
| **Plasmids** |  | **Source/Reference** |
| ZntA-mCherry | pDM1044-*zntA*, Hyg^r^ | [33] |
| ZntB-mCherry | pDM1044-*zntB*, Hyg^r^ | [33] |
| ZntC-mCherry | pDM1044-*zntC*, Hyg^r^ | [33] |
| ZntD-mCherry | pDM1044-*zntD*, Hyg^r^ | [33] |
| AmtA-mCherry | pDM1044-*amtA*, Hyg^r^ | [36] |
| mCherry-Plin | pDM1042-*plin*, Hyg^r^ | This study |

| S3B Table. *M. marinum* material used for this study | | |
| --- | --- | --- |
| Strains | **Relevant characteristics** | **Source/Reference** |
| M | wt, parental strain | L. Ramakrishnan  (University of Cambridge) |
| ΔRD1 | RD1 locus deletion mutant | L. Ramakrishnan  (University of Cambridge) [62] |
| Δ*ctpC*::Hyg^r^ | *ctpC* deletion mutant, Hyg^r^ | This study |
| Δ*ctpC* | *ctpC* deletion mutant without resistance cassette | This study |
| Δ*ctpC*/pTec19 | unmarked Δ*ctpC* mutant/pTec19, Hyg^R^ | This study |
| Δ*ctpC*/pTec19/pHK124 | unmarked Δ*ctpC*/pTec19 overexpressing *Mtb ctpC*, Hyg^R^, Apra^R^ | This study |
|  |  |  |
| Plasmids |  | **Source/Reference** |
| pMSP12::DsRed/GFP | DsRed/GFP under control of the msp12 promoter, Kan^R^ | Addgene #30171 and #30167 [63] |
| pCherry10 | mCherry under control of the G13 promoter, Hyg^r^ | Addgene #24664 [64] |
| pMV306hsp+LuxG13 | Luciferase under control of the G13 promoter, Kan^R^ | Addgene # 26161 [65] |
| pTec19 | E2-Crimson production under control of the msp12 promoter, Hyg^R^ | Addgene # 30178,  (Takaki *et al.*,2013) |
| phAE159 | shuttle phasmid for mycobacterial KO constructions, derivative of TM4 phage, Amp^r^ | [66] |
| phAE7.1 | Phage DNA containing γδ-*res* for resistance cassette removal, Kan^r^ | [66] |
| p0004S | containing γδ*res*-*sacB*-*hyg*-γδ*res* cassette comprising *sacB* and *hyg* genes flanked by the *res*-sites of γδ-resolvase, Hyg^r^ | W. Jacobs [19] |
| pHK42  p-*ctpC*-S | *ctpC* flanking regions into p0004S arms, Hyg^r^ | This study |
| pHK50  phAE159::Δ*ctpC* | *ctpC* KO phasmid, Hyg^r^ | This study |
| pHK124 pFLAG::*Rv3270* | *ctpC* into pFLAG-*SapI*, Apra^R^ | This study |

| Primers | Sequence (5’-3’) | Purpose |
| --- | --- | --- |
| oHK143  ctpC-L-Fwd-AlwNI | TTTTTCAGAAACTGCCCCGAATCACCTATTAC | Amplification of upstream AES |
| oHK144  ctpC-L-Rev-AlwNI | TTTTTCAGTTCCTGTGCTGGGACCTCTTTAAC | Amplification of upstream AES |
| oHK145  ctpC-R-Fwd-Van91I | TTTTTCCATAGATTGGCTACGGTATGTCGATCGC | Amplification of downstream AES |
| oHK146  ctpC-R-Rev-Van91I | TTTTTCCATCTTTTGGAGAATTACTACCGCCCAG | Amplification of downstream AES |
| oHK159  ctpC-L-ext | TTTTTGAGCAGAACGAAGGCAAGA | Verification of gene deletion |
| oHK160  ctpC-R-ext | TTTTTCGTTGAGAAATGGGTTGG | Verification of gene deletion |
| oHK33  p0004S-L-fwd | GGAAGTCAACAAAAAGCAAG | Sequencing |
| oHK36 p0004S-R-rev | TGGTAGCGGTGGTTTTTTTGT | Sequencing |
| oHK375 Rv3270-*SapI*-Fwd; pFLAG-*SapI* | ATATATGCTCTTCTAGTACCCTGGAAGTGGTATCGGACGCGGCC | Complementation of *ctpC* using pFLAG-*SapI* |
| oHK376 Rv3270-*SapI*-Rev; pFLAG-*SapI* | TATATAGCTCTTCATGCACGGTCCAGGCGGTAGCGGATCAACC | Complementation of *ctpC* using pFLAG-*SapI* |
| oHK328 | TTTGTCCTCCCTATCAGT | pFLAG fwd sequencing |
| oHK340 | CTTGTCATCGTCATCCT | pFLAG rev sequencing |
| oHK377 | CGTCCTGTGGTTGCTCAA | Rv3270 fwd sequencing |
| ctpC-F | tgacgacaccaccgacctggt | RT-qPCR |
| ctpC-R | taggcgtggacgacccgtac | RT-qPCR |
| ZntA-Mm-F | tgcacgtgagcgggtttggtg | RT-qPCR |
| ZntA-Mm-R | cttcagcggtgtcgcaatgttgc | RT-qPCR |
